# *JAB1* deletion in oligodendrocytes causes senescence-induced inflammation and neurodegeneration in mice

**DOI:** 10.1172/JCI145071

**Published:** 2022-02-01

**Authors:** Cristina Rivellini, Emanuela Porrello, Giorgia Dina, Simona Mrakic-Sposta, Alessandra Vezzoli, Marco Bacigaluppi, Giorgia Serena Gullotta, Linda Chaabane, Letizia Leocani, Silvia Marenna, Emanuela Colombo, Cinthia Farina, Jia Newcombe, Klaus-Armin Nave, Ruggero Pardi, Angelo Quattrini, Stefano C. Previtali

**Affiliations:** 1 Institute of Experimental Neurology (INSPE), Division of Neuroscience, IRCCS San Raffaele Scientific Institute, Milan, Italy.; 2 Institute of Clinical Physiology National Research Council (IFC-CNR), Milan, Italy.; 3 Experimental Imaging Center (CIS), IRCCS San Raffaele Scientific Institute, Milan, Italy.; 4 University Vita-Salute San Raffaele, Milan, Italy.; 5 NeuroResource, Department of Neuroinflammation, UCL Queen Square Institute of Neurology, London, United Kingdom.; 6 Department of Neurogenetics, Max Planck Institute of Experimental Medicine, Göttingen, Germany.; 7 Division of Immunology, Transplantation, and Infectious Disease, IRCCS San Raffaele Scientific Institute, Milan, Italy.

**Keywords:** Inflammation, Neuroscience, Cellular senescence, Demyelinating disorders, Mouse models

## Abstract

Oligodendrocytes are the primary target of demyelinating disorders, and progressive neurodegenerative changes may evolve in the CNS. DNA damage and oxidative stress are considered key pathogenic events, but the underlying molecular mechanisms remain unclear. Moreover, animal models do not fully recapitulate human diseases, complicating the path to effective treatments. Here we report that mice with cell-autonomous deletion of the nuclear COP9 signalosome component *CSN5* (JAB1) in oligodendrocytes develop DNA damage and defective DNA repair in myelinating glial cells. Interestingly, oligodendrocytes lacking JAB1 expression underwent a senescence-like phenotype that fostered chronic inflammation and oxidative stress. These mutants developed progressive CNS demyelination, microglia inflammation, and neurodegeneration, with severe motor deficits and premature death. Notably, blocking microglia inflammation did not prevent neurodegeneration, whereas the deletion of p21^CIP1^ but not p16^INK4a^ pathway ameliorated the disease. We suggest that senescence is key to sustaining neurodegeneration in demyelinating disorders and may be considered a potential therapeutic target.

## Graphical abstract

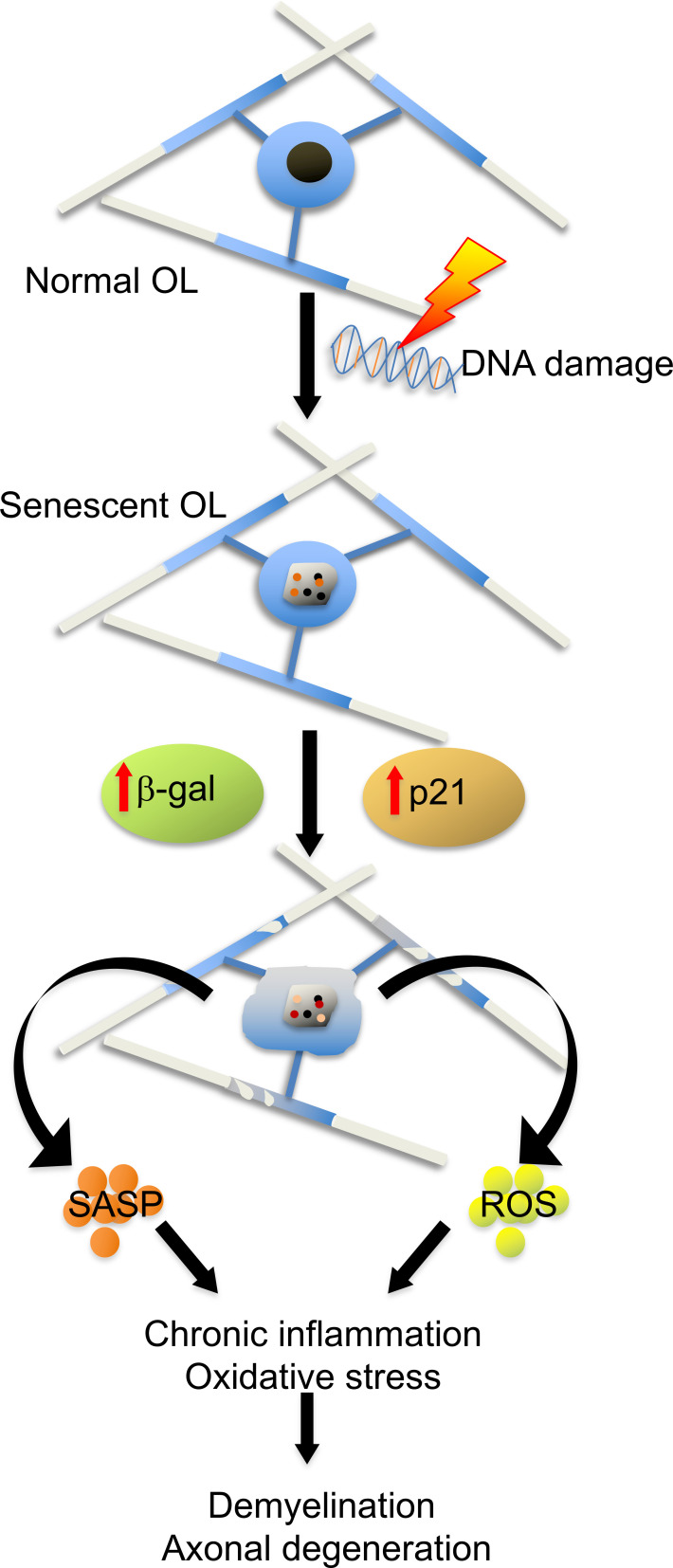

## Introduction

Myelin is a highly specialized protein- and lipid-rich membrane that wraps around axons in the central and peripheral nervous systems (CNS and PNS). It is made by oligodendrocytes in the CNS and Schwann cells in the PNS. The main function of myelin is to enable energy-efficient saltatory conduction (1), though recent studies have highlighted its critical importance in neuronal survival and in providing trophic support for axon integrity (2). Accordingly, white matter disorders in which mature or myelinating oligodendrocytes are damaged can evolve into devastating neurodegenerative disorders, such as multiple sclerosis (MS) and leukodystrophies (3, 4). Thus, a better understanding of the processes regulating oligodendrocyte myelination, maintenance, and axon-glia interaction is crucial to unravel mechanisms underlying neurodegeneration and to develop potential treatments.

Oligodendrocytes are the primary target of white matter disorders, but it is still unclear how this damage is related to axonal loss. Although demyelination is considered the primary cause of axonal degeneration, axonal damage may even precede or parallel demyelination (5, 6). Indeed, demyelination per se does not necessarily result in axonal damage (7). Oligodendrocytes are particularly sensitive to DNA damage and oxidative stress, events that have been associated with progressive axonal loss and are considered to be key elements in MS pathology and many neurodegenerative disorders (8–11). Despite their critical role, it is still unclear how DNA damage and oxidative stress are molecularly linked and can foster progressive axonal loss.

Jun activation domain–binding protein 1 (JAB1; or CSN5) is a multifunctional nuclear protein involved in controlling several aspects of cell function through gene transcription and protein degradation (12). Notably, along with the control of cell cycle progression (12, 13), JAB1 regulates DNA damage and repair (14, 15). JAB1 is the catalytic subunit of the constitutive photomorphogenesis 9 (COP9) signalosome (CSN), a modifier of the cullin-RING ubiquitin ligase (CRL) complexes. The main CSN function is the enzymatic removal of NEDD8 from the cullin component of the ligase, a requirement for proper functioning of CRL complexes. CRL-controlled substrates (e.g., MYC, p27^KIP1^, β-catenin, cyclin E) are key regulators of cell cycle progression and DNA repair (16).

We previously reported that in the PNS, JAB1 autonomously controls Schwann cell–axon interaction and its loss results in demyelination and axonal degeneration of peripheral nerves (17). Since oligodendrocytes are sensitive to DNA damage, oxidative stress, and perturbation of cell cycle progression, we now investigated whether JAB1 in the oligodendrocyte lineage might control CNS myelination and maintenance. Here we show that oligodendrocyte conditional deletion of *Jab1* — with 2 different transgenes — causes progressive CNS demyelination, inflammation, and axonal degeneration. Rescue of the inflammatory response did not prevent neurodegeneration. The molecular mechanism was shown to reside in the activation of the senescence pathway by DNA damage in mature oligodendrocytes.

## Results

### JAB1 expressed in oligodendrocytes is required for oligodendrocyte growth and survival.

**Figure 1:**
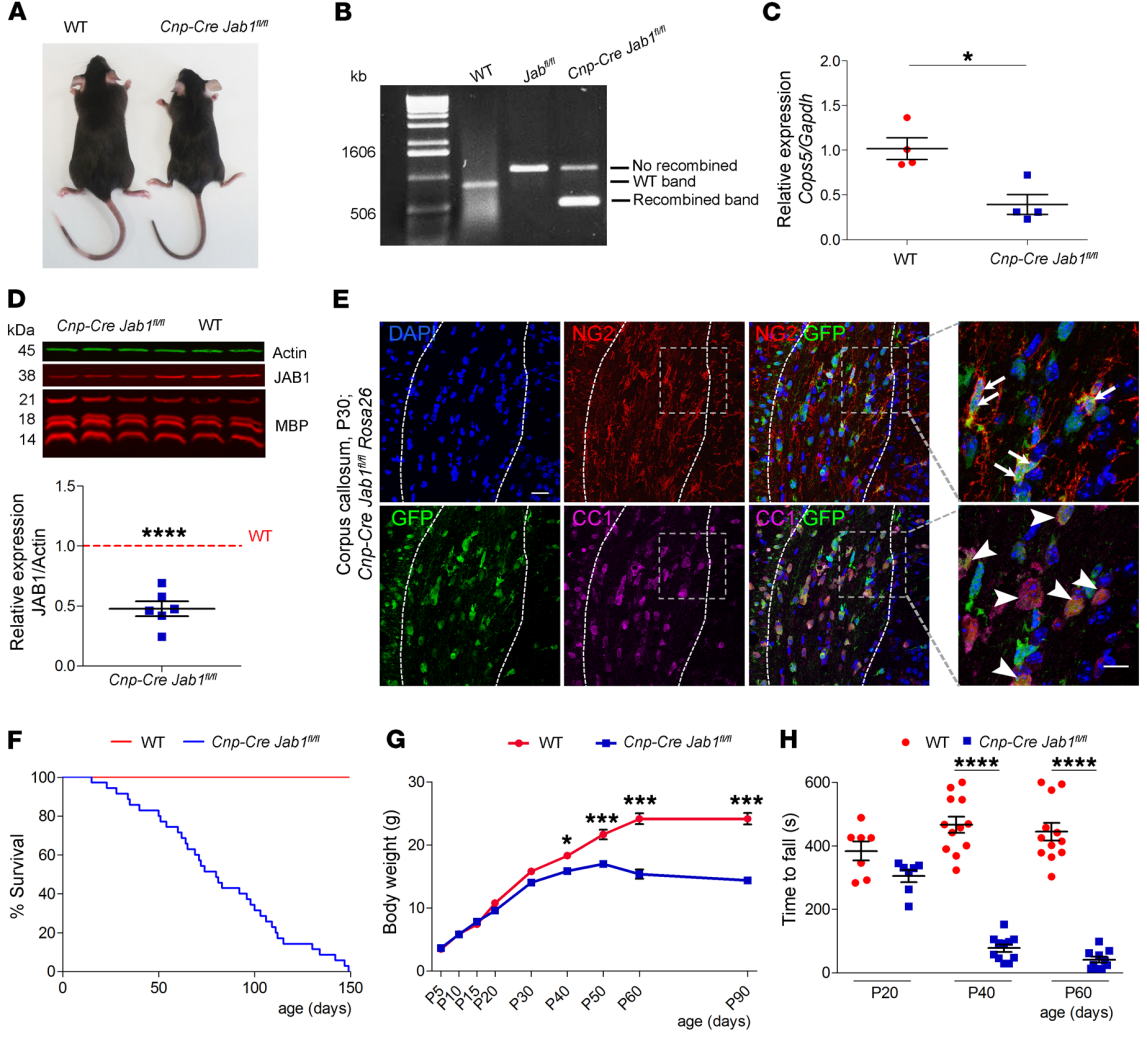
Generation of oligodendrocyte-conditional *Jab1*-null mice. (**A**) *Cnp-Cre Jab1^fl/fl^* mouse showing reduced size as compared with littermate control. (**B**) Genotyping PCR using optic nerve–derived DNA providing genomic recombination only in *Cnp-Cre Jab1^fl/fl^* mice. (**C**) qPCR for *Cops5* (*Jab1*) expression in sorted oligodendrocytes (O1^+^) showing reduced expression in *Cnp-Cre Jab1^fl/fl^* as compared with WT mice (*P < 0.05; *n =* 4; 2-tailed nonparametric Mann-Whitney *U* test). (**D**) Western blot analysis and quantification of optic nerve homogenate showing JAB1 reduction in *Cnp-Cre Jab1^fl/fl^* mice (****P < 0.0001; *n =* 6; 1-sample 2-tailed Student’s *t* test; WT is reported as equal to 1). (**E**) Confocal images of the corpus callosum (within dashed lines) of *Cnp-Cre Jab1^fl/fl^ Rosa26* mice stained for GFP, CC1, NG2, and DAPI. Recombined OPCs positive for GFP and NG2 (arrows) and oligodendrocytes positive for GFP and CC1 (arrowheads) are labeled in the inset magnification. (**F**) Kaplan-Meier curve showing reduced survival in mutant mice (*n =* 100 controls and 72 mutants; ****P < 0.0001, χ^2^ Mantel-Cox test). (**G**) Mutant mice showing significant body weight reduction over time (*P < 0.05, ***P < 0.001; *n =* 10; 2-way ANOVA with Bonferroni’s post hoc correction). (**H**) Rotarod analysis showing significant motor deficits in mutant mice since P40 (****P < 0.0001; 2-tailed nonparametric Mann-Whitney *U* test). Scale bar: 20 μm; inset, 10 μm.

To define the role of JAB1 in myelin formation, we generated mutant mice specifically lacking its expression in oligodendrocytes. Thus, we crossed *Jab1^fl/fl^* mice with *Cnp1-Cre* transgene mice (Figure 1, A and B, and ref. 18). The latter is active in the late phase of oligodendrocyte precursor (OPC) development and in differentiated oligodendrocytes, and ensures efficient recombination (19, 20). O1^+^ FACS-sorted oligodendrocytes from the mutant mouse brains showed significant reduction of *Jab1* mRNA (Figure 1C, and Supplemental Figure 1A for gating strategy of FACS; supplemental material available online with this article; https://doi.org/10.1172/JCI145071DS1). In addition, JAB1 protein was significantly reduced in Western blot analysis in mutant optic nerves (Figure 1D). Moreover, breeding of *Cnp-Cre Jab1^fl/fl^* with *EYFP-Rosa26* mice revealed high rates of recombination: 58% in NG2^+^ OPCs and 80% in CC1^+^ oligodendrocytes (Figure 1E).

*Cnp-Cre Jab1^fl/fl^* mice appeared phenotypically normal until postnatal day 20 (P20). After P40, mutant mice showed progressive motor impairment (tremor, unsteady gait) along with reduced size and weight as compared with controls (Figure 1G). Remarkably, all *Jab1*-mutant mice died by P150, and already at P70 we observed 50% mortality in the colony (Figure 1F).

We also measured motor performances (strength and coordination) by rotarod analysis. We observed normal function at P20, but significant impairment in *Cnp-Cre Jab1^fl/fl^* mice from P40 (Figure 1H).

### Jab1-mutant mice are characterized by progressive CNS demyelination and axonal loss.

**Figure 2:**
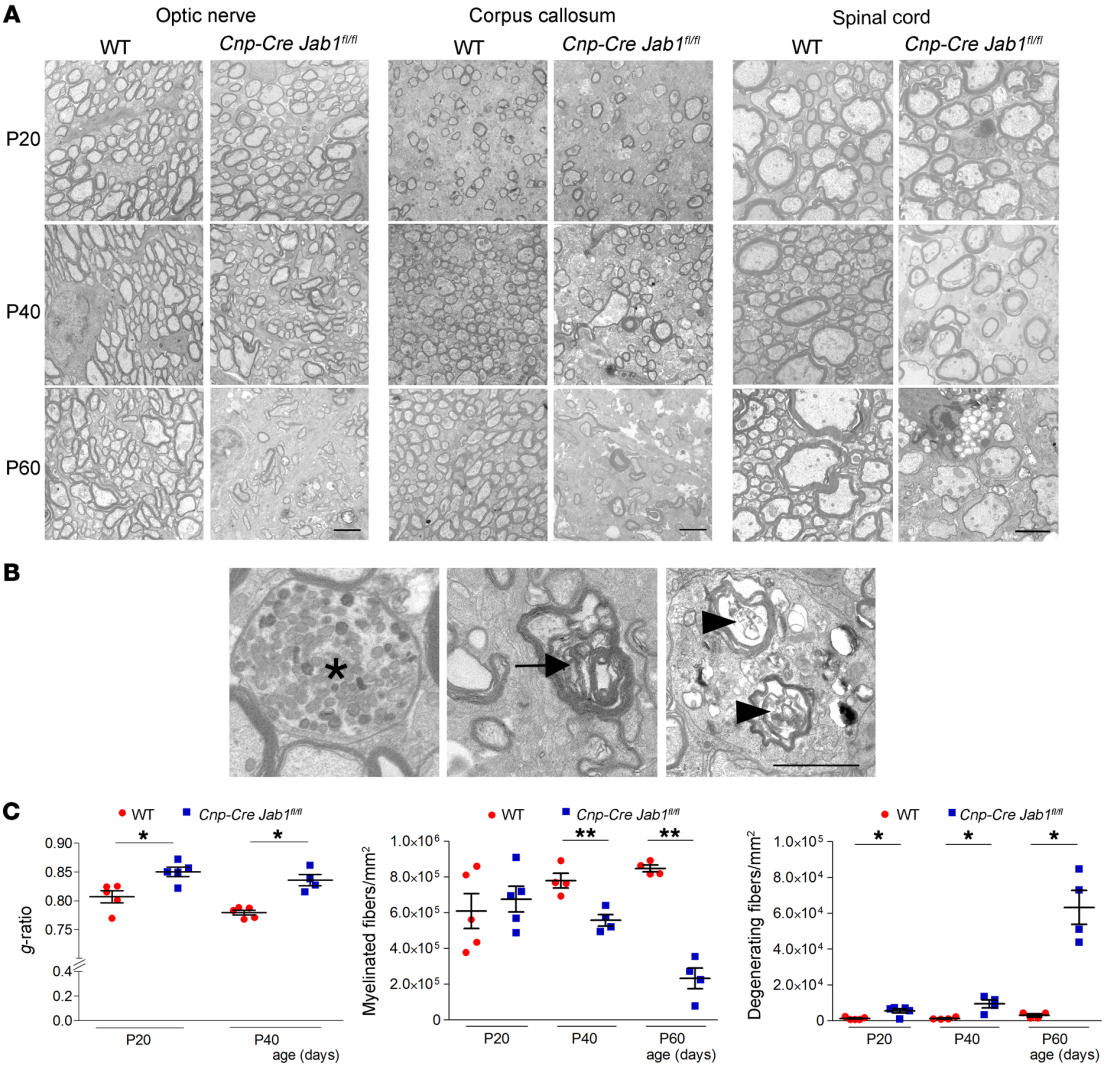
Morphological abnormalities in *Jab1*-mutant mice. (**A**) Electron micrographs of transverse sections of the optic nerve, corpus callosum, and spinal cord from WT and *Cnp-Cre Jab1^fl/fl^* mice at ages P20, P40, and P60, showing progressive demyelination and axonal loss. (**B**) Representative electron micrographs of spinal cord cross sections from P60 *Cnp-Cre Jab1^fl/fl^* mice, showing axonal spheroid (asterisk), swelling and accumulation of organelles and ovoids (arrow), and myelin fragmentation (arrowheads). (**C**) Quantification of *g*-ratio and numbers of myelinated fibers and degenerating fibers in the optic nerves from WT and *Cnp-Cre Jab1^fl/fl^* mice at different time points (*P < 0.05, **P < 0.01; *n =* 4–5; at least 2000 fibers counted per mouse; 2-tailed nonparametric Mann-Whitney *U* test). Scale bars: (**A** and **B**) 2 μm.

In agreement with their phenotypic appearance, P20 *Cnp-Cre Jab1^fl/fl^* mice were slightly hypomyelinated in optic nerves, with morphologically normal myelin sheath and no alterations in the number of fibers (Figure 2, A and C, and Supplemental Figure 2A). Accordingly, the amounts of myelin basic protein (MBP) and myelin-associated glycoprotein (MAG) were comparable to those in controls in P20 optic nerves, corpus callosum, and spinal cord (Supplemental Figure 2, B and C, and data not shown).

From P40 onward, we observed progressive demyelination in optic nerve, corpus callosum, and spinal cord of *Cnp-Cre Jab1^fl/fl^* mice (Figure 2A), as corroborated by increased *g*-ratio values (Figure 2C) and consistent reduction in myelin protein expression (Supplemental Figure 2, B and C). At P60, almost all fibers in the CNS were devoid of myelin (Figure 2A). Notably, starting at P40, *Cnp-Cre Jab1^fl/fl^* mice also showed progressive neurodegeneration (Figure 2A), with signs of active axonal degeneration (Figure 2B) and significant loss of myelinated axons (Figure 2C).

To corroborate findings of axonal degeneration, we evaluated ganglion cells as major output cells of the retina to form the optic nerve. We stained the retina with anti-BRN3A antibody to identify ganglion cells, and we quantified cell numbers at different time points. We did not observe significant differences at P20 and P40, while the number of ganglion cells was significantly reduced in *Cnp-Cre Jab1^fl/fl^* mice at P60 (Supplemental Figure 3, A and B). Moreover, we examined levels of non-phosphorylated neurofilaments as a marker of axonal degeneration in the brain, as previously reported (21, 22). As depicted in Supplemental Figure 3, C and D, we observed a significant increase in the amount of non-phosphorylated neurofilaments in brain homogenates of *Cnp-Cre Jab1^fl/fl^* as compared with WT mice.

Finally, as *Cnp-Cre* transgene also recombines in Schwann cells, we evaluated peripheral nerves of mutant mice. As previously reported with *P0-Cre* transgene, which recombines specifically in Schwann cells (17), *Cnp-Cre Jab1^fl/fl^* mice also developed a dysmyelinating neuropathy with axonal sorting defects (Supplemental Figure 4A). As peripheral neuropathy may influence rotarod analysis, we compared P60 *Cnp-Cre Jab1^fl/fl^* with *P0-Cre Jab1^fl/fl^* mice, revealing that motor deficit was significantly worse in *Cnp-Cre Jab1^fl/fl^* mice as a consequence of CNS neurodegeneration (Supplemental Figure 4B).

### Functional analyses in Jab1-mutant mice.

**Figure 3:**
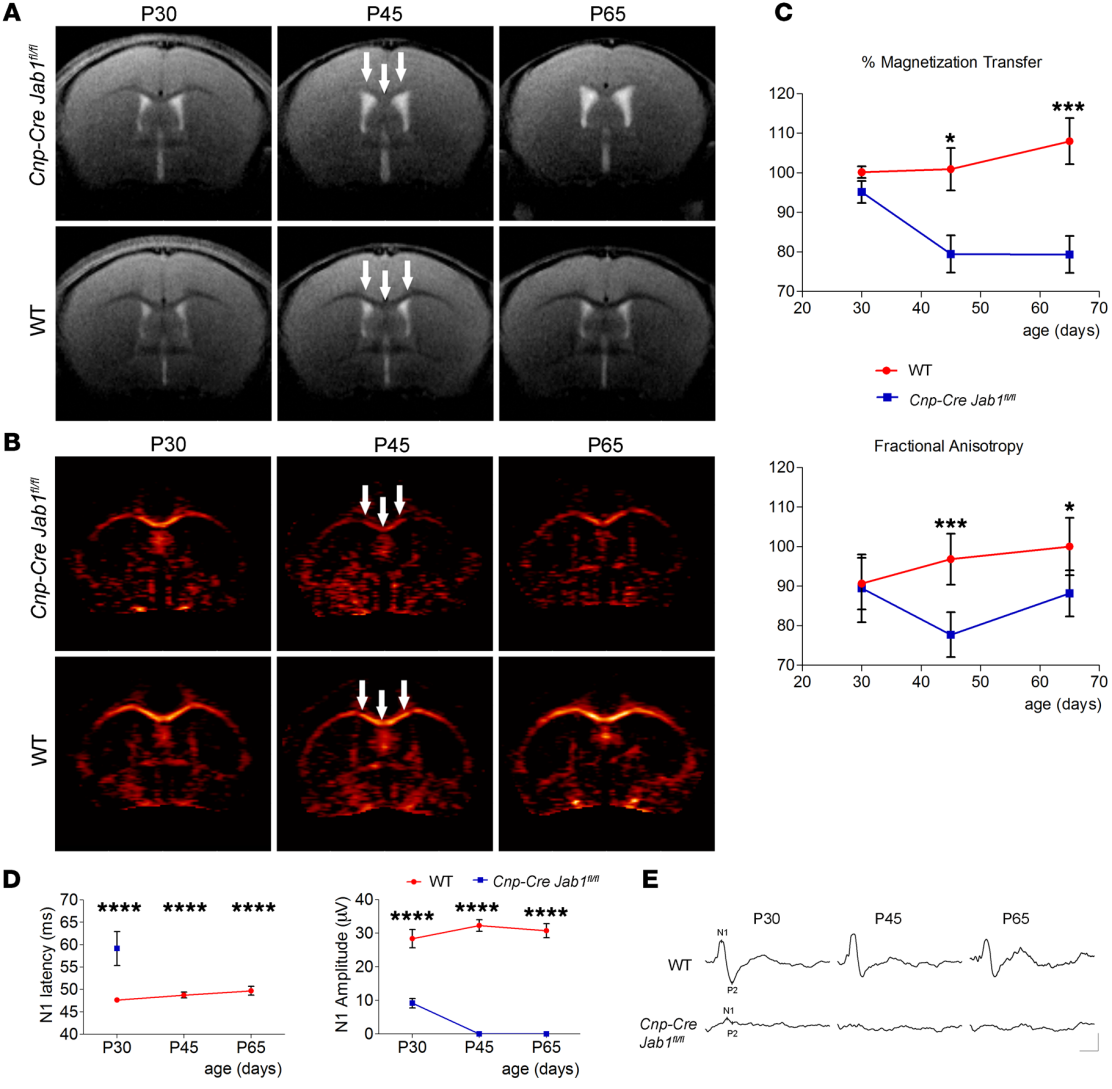
Longitudinal MRI and VEP evaluation. (**A**) Brain MT MRI images of WT and *Cnp-Cre Jab1^fl/fl^* mice at different ages (P30, P45, and P65). While in WT mice, the corpus callosum is clearly delineated on MT images (in dark, arrows), it is hardly distinguishable in *Jab1*-mutant mice, in which the contrast is similar at the first exam (P30) and becomes hyperintense at later stages (P45 and P60, arrows). (**B**) With DTI (color map of FA), the corpus callosum is well discriminated in all the animals, but the FA (color map) is clearly decreased at P45 and P60 in *Jab1*-mutant as compared with WT mice (arrows). (**C**) Quantitative analysis of MT and FA measured in the medial of the corpus callosum. Both MT and FA were significantly reduced with age in *Jab1*-mutant mice as compared with healthy controls (*P < 0.05, ***P < 0.001; *n =* 6; 2-way ANOVA with Bonferroni’s post hoc correction). (**D**) N1 latency and amplitude measured in WT and *Cnp-Cre Jab1^fl/fl^* mice at age P30, P45, and P65 (****P < 0.0001; *n =* 7 WT, 6 mutant; 2-way ANOVA with Bonferroni’s post hoc correction). (**E**) Representative VEP waveforms recorded from WT and *Cnp-Cre Jab1^fl/fl^* at different ages. Horizontal bar, 50 ms; vertical bar, 20 μV.

To further evaluate demyelination and neurodegeneration, we performed MRI using magnetization transfer (MT) contrast method to estimate myelination and diffusion tensor imaging (DTI) to measure the fractional anisotropy (FA) to evaluate axonal integrity. At P30, *Cnp-Cre Jab1^fl/fl^* mice were almost the same as controls (Figure 3, A–C). However, at P45 the corpus callosum of mutant mice showed significant hyperintense signals in MT sequences, which were even more pronounced at P65 (Figure 3, A and C). Similarly, on DTI the normal control corpus callosum was well defined, but we observed a significant reduction in FA (both mean and axial diffusivity) in mutant mice at P45 and P65 (Figure 3, B and C). These changes correlated with progressive demyelination and axonal loss in the corpus callosum of *Cnp-Cre Jab1^fl/fl^* mice.

Finally, we performed visual evoked potentials (VEPs) to functionally test optic nerve activity. At P30, *Cnp-Cre Jab1^fl/fl^* mice showed detectable VEPs with significantly increased latency and reduced amplitude (Figure 3, D and E), and at P45 and P60, both latency and amplitude could not be detected in mutant mice (Figure 3, D and E), suggesting severe functional deficit.

### CNS degeneration in Cnp-Cre Jab1^fl/fl^ mice is associated with inflammation.

**Figure 4:**
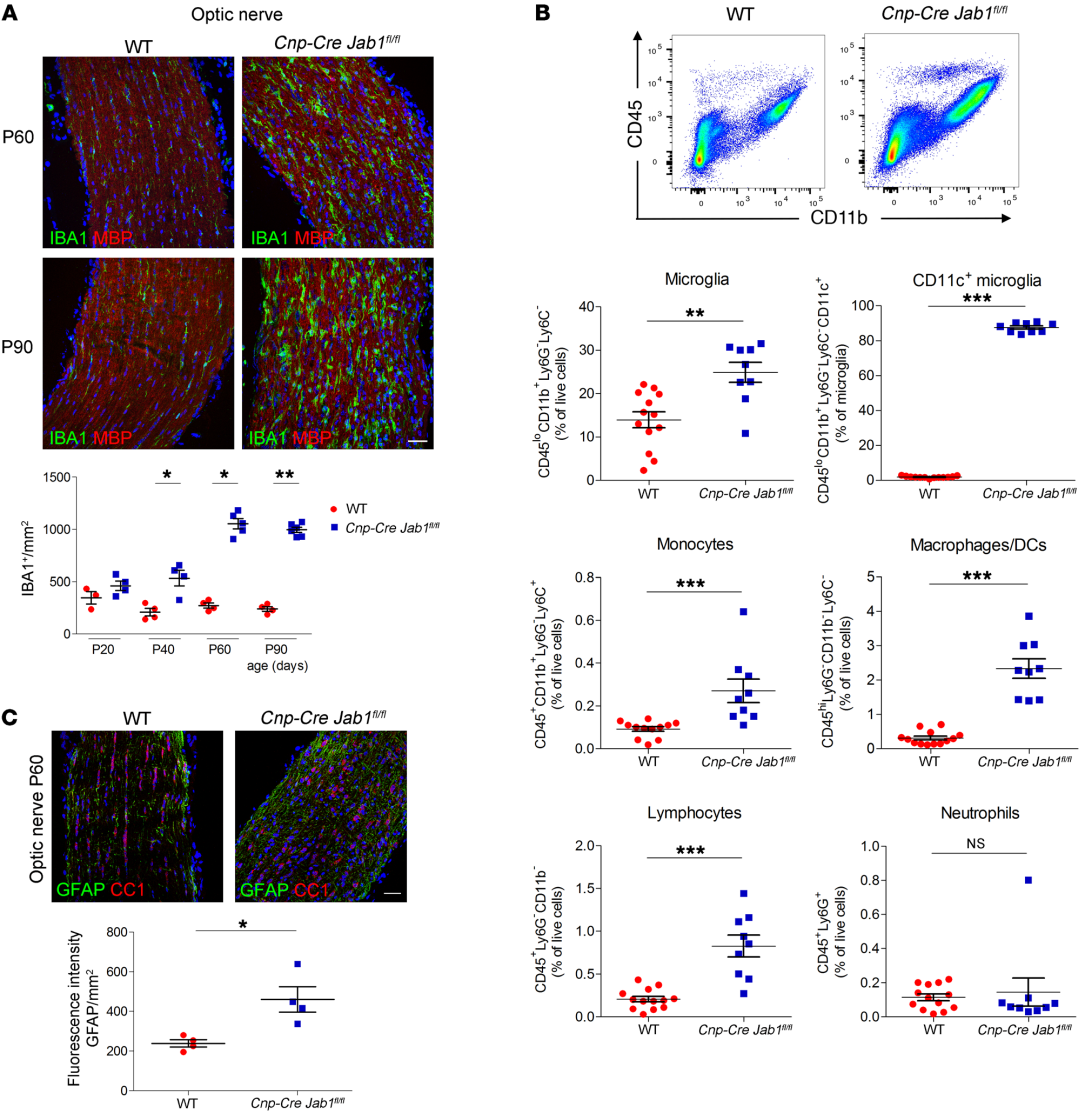
Inflammatory infiltration in *Jab1*-mutant mice. (**A**) Immunolabeling of WT and *Cnp-Cre Jab1^fl/fl^* optic nerves for IBA1 and MBP at ages P60 and P90 and relative quantification from P20 to P90, showing progressive increase in the number of IBA1^+^ cells in mutant mice (*P < 0.05, **P < 0.01; *n =* 3–5; 2-tailed nonparametric Mann-Whitney *U* test). (**B**) FACS analysis of P60 WT and *Cnp-Cre Jab1^fl/fl^* brain for CD45, CD11b (representative plots), CD11c, Ly6G, and Ly6C, showing quantification of microglia, activated microglia, lymphocytes, monocytes, macrophages, and neutrophils (**P < 0.01, ***P < 0.001; *n =* 13 WT, *n =* 9 *Cnp-Cre Jab1^fl/fl^*; 2-tailed nonparametric Mann-Whitney *U* test). (**C**) Immunolabeling for GFAP and CC1 of the optic nerve from WT and *Cnp-Cre Jab1^fl/fl^* mice at age P60 and relative quantification, showing increased number of GFAP^+^ reactive astrocytes in mutant mice (*P < 0.05; *n =* 4; 2-tailed nonparametric Mann-Whitney *U* test). Scale bars: (**A** and **C**) 40 μm.

Between P40 and P60, in addition to demyelination and neurodegeneration, we observed in *Cnp-Cre Jab1^fl/fl^* mice a diffuse microglial/macrophage inflammatory infiltrate, as revealed by anti-IBA1 staining (Figure 4A). To further characterize the inflammatory infiltrate, we FACS-sorted inflammatory cells from brains. We confirmed that mutant mice had a significant increase both in microglia (CD45^lo^CD11b^+^Ly6G^–^Ly6C^–^) and in macrophages (CD45^hi^CD11b^+^Ly6G^–^Ly6C^–^), which represented the majority of the inflammatory cells (Figure 4B and Supplemental Figure 1B). Interestingly, in *Cnp-Cre Jab1^fl/fl^* mice, microglia displayed high CD11c positivity, were smaller in size (forward scatter, FSC-A MFI), and had higher granularity (SSC-A MFI), all hallmarks of activation (Figure 4B and Supplemental Figure 1C). Although significantly increased in number, lymphocytes and monocytes represented a smaller proportion of the inflammatory cells, whereas neutrophils did not differ from controls (Figure 4B and Supplemental Figure 1B). In addition, we also observed moderate astrogliosis, as glial fibrillary acidic protein (GFAP) staining was increased in optic nerves of *Jab1*-mutant mice (Figure 4C).

### Inhibition of monocyte recruitment or depletion of microglia is not sufficient to rescue neurodegeneration in Cnp-Cre Jab1^fl/fl^ mice.

**Figure 5:**
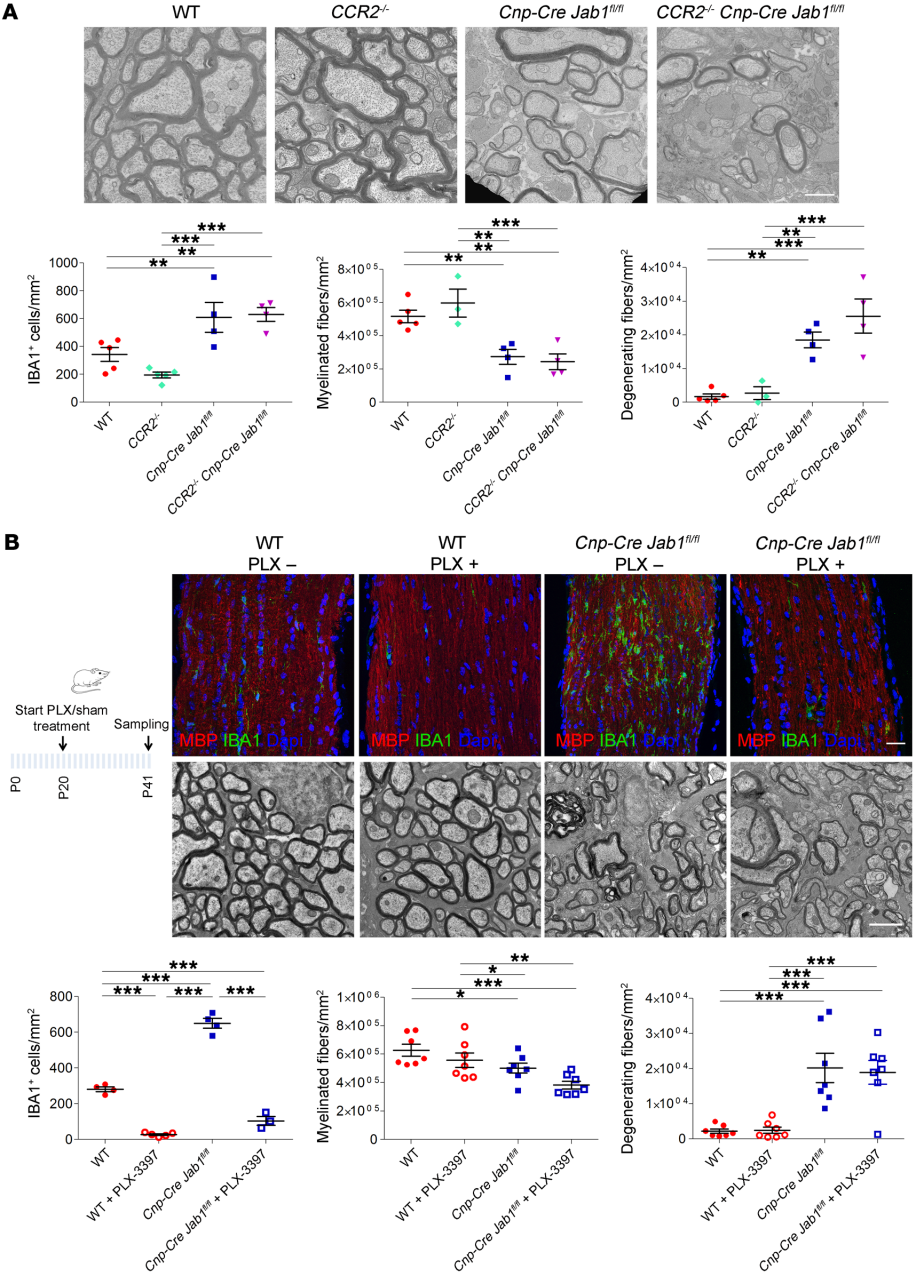
Depletion of microglia activation does not ameliorate neurodegeneration in *Jab1*-mutant mice. (**A**) Electron micrographs of transverse sections of P50 optic nerve from WT, *CCR2^–/–^*, *Cnp-Cre Jab1^fl/fl^*, and *Cnp-Cre Jab1^fl/fl^* *CCR2^–/–^* mice and relative quantification of IBA1^+^ cells and numbers of myelinated fibers and degenerating fibers, showing no significant differences between *Jab1^–/–^* and double-mutant *Jab1^–/–^* *CCR2^–/–^* mice (**P < 0.01, ***P < 0.001; *n =* 4–5; 1-way ANOVA with Bonferroni’s multiple-comparison test). (**B**) Confocal images (for IBA1 and MBP) and electron micrographs of P41 optic nerve from WT and *Cnp-Cre Jab1^fl/fl^* mice treated (PLX +) or not (PLX –) for 21 days with PLX3397 (see schematic) and relative quantification of IBA1^+^ cells and numbers of myelinated and degenerating fibers. Although the number of IBA1^+^ cells was significantly reduced in PLX-treated mice, no differences in the numbers of myelinated and degenerating fibers were observed (*P < 0.05, **P < 0.01, ***P < 0.001; *n =* 4–9; 1-way ANOVA with Bonferroni’s multiple-comparison test). Scale bars: (**A**) 1 μm; (**B**) immunohistochemistry, 40 μm; electron microscopy, 2 μm.

To determine whether the observed inflammatory infiltrate could contribute to demyelination and neurodegeneration and to discriminate the role of monocytes/macrophages versus microglia, we first crossed C-C chemokine receptor type 2–knockout (*Ccr2^–/–^*) with *Jab1*-mutant mice. CCR2 is in fact required for the egress of monocytes from the bone marrow and has a prevalent role in monocyte/macrophage recruitment. Furthermore, *Ccr2^–/–^* mice are resistant to experimental autoimmune encephalitis (23, 24). Indeed, P50 *Cnp-Cre Jab1^fl/fl^ Ccr2^–/–^* mutants did not show differences from *Cnp-Cre Jab1^fl/fl^* mice in the numbers of inflammatory cells, myelinated fibers, and degenerating fibers (Figure 5A).

Since inhibition of monocyte/macrophage recruitment in *Cnp-Cre Jab1^fl/fl^* brains did not seem responsible for the observed neurodegeneration, we next explored the role of microglia. To test this hypothesis, we depleted microglia in *Cnp-Cre Jab1^fl/fl^* mice by using PLX3397, a selective inhibitor of colony-stimulating factor 1 receptor (CSF1R) able to eradicate CNS microglia (25, 26). Although PLX3397 treatment resulted in a significant reduction in IBA1^+^ microglia in both *Cnp-Cre Jab1^fl/fl^* and control optic nerves, we did not observe any amelioration in the number of myelinated or degenerating axons in mutant mice (Figure 5B).

Based on these results, we posit that neurodegeneration in *Cnp-Cre Jab1^fl/fl^* mice is not due to an overt role of microglia and monocytes, since its inhibition does not ameliorate disease course.

### Jab1-mutant oligodendrocytes develop and differentiate normally.

We previously reported that in the PNS JAB1 controls p27^KIP1^ levels in Schwann cells and their cell cycle progression, differentiation, and survival (17). Thus, we next determined whether the degeneration in *Cnp-Cre Jab1^fl/fl^* mice could be the result of reduced oligodendrocyte number, survival, and/or differentiation.

We first counted the total number of cells in optic nerves from P20 to P90. We did not observe any difference until P60, when this was increased in *Jab1*-mutant mice (Supplemental Figure 5A), most likely because of the inflammatory infiltrates. Next, we specifically assessed the number of OPCs (NG2^+^) and mature oligodendrocytes (CC1^+^) in optic nerves. While the number of mature oligodendrocytes did not change at any time point analyzed, OPCs were similar to control until P60 and then increased (Supplemental Figure 5B), suggesting that JAB1 deletion did not affect cell cycle progression and/or the proliferation/survival ratio.

To determine whether in the absence of JAB1, OPC/oligodendrocyte proliferation was affected, we performed BrdU incorporation assay and Ki67 staining in optic nerves. Whereas at P20, BrdU incorporation was comparable to controls, we observed an overall significant increase in all cells and in NG2^+^ OPCs in P60 optic nerves of mutant mice (Supplemental Figure 5C), as confirmed also by Ki67 staining (Supplemental Figure 5D). Next, we investigated whether JAB1 ablation might lead to altered survival in the oligodendrocyte lineage. Caspase-3 staining in P40 and P60 optic nerves did not show any significant difference between mutants and controls, while at P90 we observed a significant number of apoptotic oligodendrocytes in mutant nerves (Supplemental Figure 5E).

Finally, we investigated whether JAB1 might control OPC terminal differentiation. We injected BrdU for 3 consecutive days in P20 mice that were then analyzed at P30 (Supplemental Figure 5F). Quantification of the number of double-positive BrdU/CC1 oligodendrocytes at P30 was similar in *Jab1*-mutant mice and controls (Supplemental Figure 5F), suggesting that OPCs differentiate normally into mature oligodendrocytes.

Collectively, these results suggest that, in contrast to Schwann cells, JAB1 is not required for oligodendrocyte survival and differentiation. Accordingly, p27^KIP1^ levels did not change in optic nerves of *Jab1* mutants (Supplemental Figure 6).

### Jab1-mutant oligodendrocytes activate senescence-like program.

**Figure 6:**
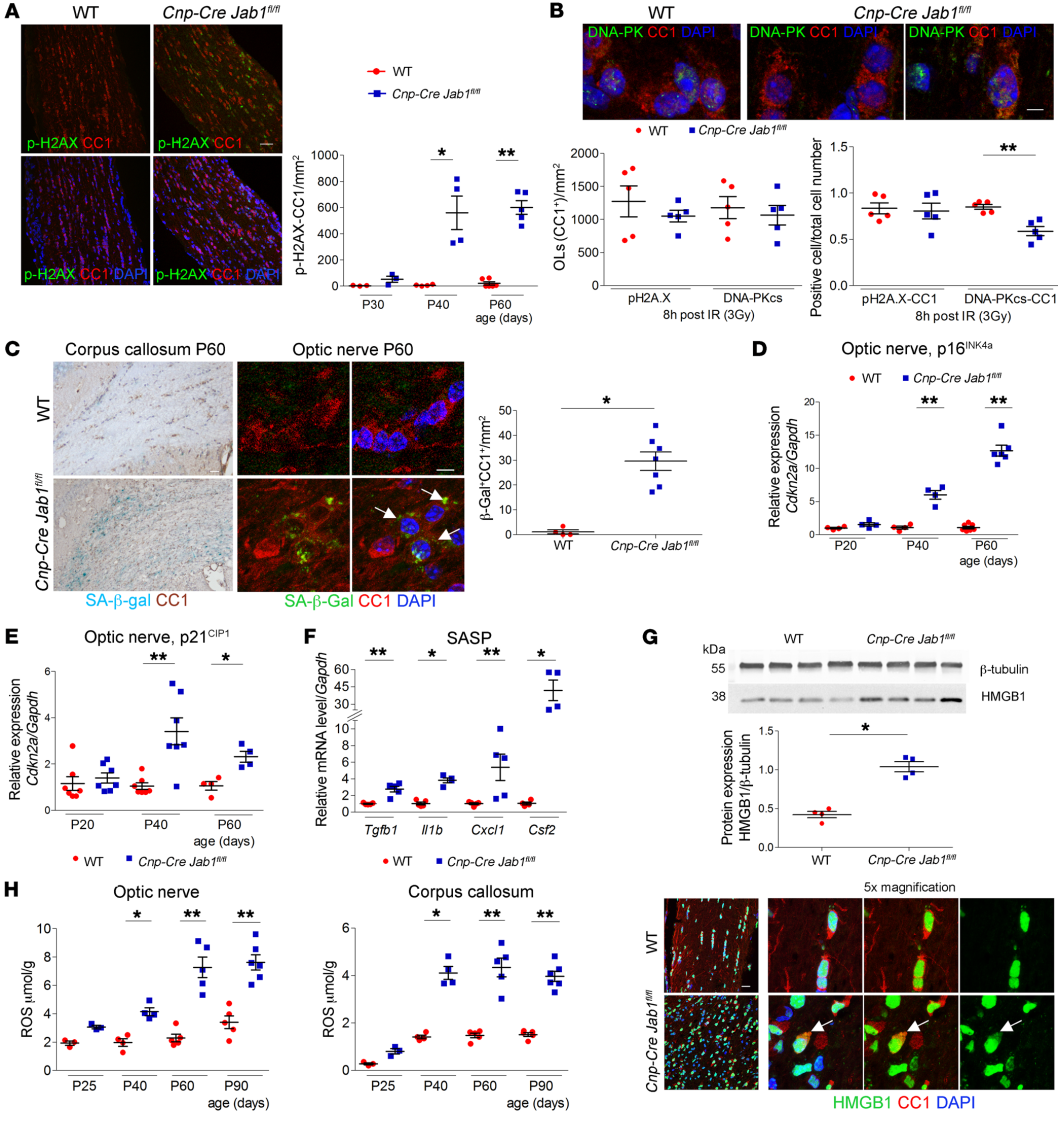
DNA damage and senescence in mutant mice. (**A**) Confocal immunolabeling of WT and mutant optic nerves stained for CC1 and p-H2AX; quantification shows significant increase of p-H2AX^+^ oligodendrocytes in mutant mice from P40 (*P < 0.05, **P < 0.01; *n =* 3–5; 2-tailed nonparametric Mann-Whitney *U* test). (**B**) Confocal images of ex vivo optic nerves x-ray–irradiated (IR) to induce DNA damage, stained for DNA-PKcs and CC1; the majority of WT oligodendrocytes (OLs) have nuclear expression of DNA-PK, whereas this percentage is reduced in *Jab1^–/–^* optic nerves (**P < 0.01; *n =* 5; 2-tailed nonparametric Mann-Whitney *U* test). (**C**) β-Gal staining and quantification of the corpus callosum and optic nerve from P60 WT and *Cnp-Cre Jab1^fl/fl^* mice double-stained with CC1, showing senescent oligodendrocytes in mutant mice (*P < 0.05; *n =* 5–7; 2-tailed nonparametric Mann-Whitney *U* test). (**D**) qPCR for *Cdkn2a* (p16^INK4a^) in the optic nerve from WT and *Cnp-Cre Jab1^fl/fl^* mice, showing significant increase from P40 (**P < 0.01; *n =* 5–6; 2-tailed nonparametric Mann-Whitney *U* test). (**E**) qPCR for *Cdkn1a* (p21^CIP1^) in the optic nerve from WT and *Cnp-Cre Jab1^fl/fl^* mice, showing significant increase from P40 (*P < 0.05, **P < 0.01; *n =* 5–6; 2-tailed nonparametric Mann-Whitney *U* test). (**F**) qPCR for SASP in the optic nerve from P60 WT and *Cnp-Cre Jab1^fl/fl^* mice, showing significant elevation in mutant mice (*P < 0.05, **P < 0.01; *n =* 4–6; 2-tailed nonparametric Mann-Whitney *U* test). (**G**) Western blot analysis, quantification, and confocal images for HMGB1 in P60 optic nerves of WT and *Cnp-Cre Jab1^fl/fl^* mice; HMGB1 is increased in mutant mice and is also translocated in the cytoplasm of mutant oligodendrocytes (arrows) (*P < 0.05; *n =* 4; 2-tailed nonparametric Mann-Whitney *U* test). (**H**) ROS quantification in optic nerve and corpus callosum from WT and *Cnp-Cre Jab1^fl/fl^* mice, showing significant ROS elevation from P40 (*P < 0.05, **P < 0.01; *n =* 3–5; 2-tailed nonparametric Mann-Whitney *U* test). Scale bars: (**A**) 40 μm; (**B**) 2 μm; (**C**) light microscopy, 50 μm; fluorescence, 2 μm; (**G**) 20 μm.

Since JAB1 has a critical role in DNA integrity and DNA damage repair (14), we next determined whether these processes are hampered in oligodendrocytes lacking JAB1 expression. Thus, we performed immunohistochemical analyses with anti–phospho–histone H2AX (anti–p-H2AX). While p-H2AX in optic nerves of control mice was virtually absent at all analyzed time points, we observed p-H2AX in a significant number of mutant oligodendrocytes, particularly at P40 and P60 (Figure 6A).

To further corroborate these results, we next investigated the nonhomologous end joining (NHEJ) mechanism of repair, which is operating in nondividing mature oligodendrocytes (27). As under physiological conditions there was no DNA damage, and to examine differences between controls and *Jab1*-mutant oligodendrocytes, we artificially induced DNA damage by x-ray irradiation. Optic nerves from control and *Cnp-Cre Jab1^fl/fl^* mice were cultured in vitro and x-ray–irradiated (3 Gy) to induce massive DNA damage. The activation of DNA-PK, the primary nuclease complex involved in NHEJ, was significantly reduced in *Cnp-Cre Jab1^fl/fl^* oligodendrocytes (Figure 6B), though a similar number of mature oligodendrocytes (CC1^+^) showed DNA damage in both *Jab1*-mutant and control optic nerves.

As a result of DNA damage, JAB1 regulates cell fate through DNA repair. In the absence of JAB1, the cell fate could vary depending on the extent of DNA damage and may result in apoptosis or in senescence (28). Since we did not observe apoptosis in *Jab1*-null oligodendrocytes until late stage (P90; Supplemental Figure 5E), we next investigated whether in mature oligodendrocytes JAB1 might control senescence. We thus evaluated specific markers such as senescence-associated β-galactosidase (SA-β-gal), p16^INK4a^, and p21^CIP1^. As expected, we observed SA-β-gal staining in oligodendrocytes already at P40 and significantly increased at P60 in optic nerve and corpus callosum of *Jab1*-mutant mice, but not in controls (Figure 6C). Accordingly, we also detected significant increases of p16^INK4a^ and of p21^CIP1^ in P40–P60 optic nerves of *Jab1*-mutant mice (Figure 6, D and E). Interestingly, p21^CIP1^ and p53 (p16^INK4a^ was not detectable; Supplemental Figure 4C) did not change in the peripheral nerve of *Cnp-Cre Jab1^fl/fl^* mice. Also, we could not detect SA-β-gal staining in Schwann cells (Supplemental Figure 4D), confirming that the neuropathy due to the lack of JAB1 is caused by a different mechanism, and that lack of JAB1 is not necessarily a common mechanism to induce senescence in the CNS and PNS.

It has been reported that senescent cells release soluble inflammatory factors (senescence-associated secretory phenotype [SASP]) and produce reactive oxygen species (ROS), which may amplify cell/tissue damage (28). Thus, we measured levels of SASP molecules in optic nerves of *Cnp-Cre Jab1^fl/fl^* mice and controls by quantitative PCR (qPCR) analyses. We observed significant increase in IL-1β, TGF-β1, chemokine (C-X-C motif) ligand 1 (CXCL1), and granulocyte-macrophage colony-stimulating factor (GM-CSF) mRNA expression specifically in mutant mice (Figure 6F). Accordingly, high-mobility group box 1 (HMGB1), a further component of the secretory pathway of senescent cells that may be involved in perpetrating senescence (29), was significantly increased in optic nerve homogenates from mutant mice (Figure 6G) and, as expected, mobilized in the cytoplasm of senescent oligodendrocytes, differently from the nuclear localization normally present in healthy cells (Figure 6G). Moreover, ROS levels were increased starting at P40 in *Jab1*-mutant optic nerves and corpus callosum (Figure 6H), and at P90 in the spinal cord (data not shown), when measured by electron paramagnetic resonance spectroscopy.

To confirm the above results and to demonstrate that SASP originated from mutant oligodendrocytes, we FACS-sorted O1^+^ oligodendrocytes from P40 brain of *Cnp-Cre Jab1^fl/fl^* and control mice and performed RNA-Seq and qPCR analyses. We observed a significant number of genes that were downregulated (930 genes) or upregulated (1576; Supplemental Figure 7A and Supplemental Table 1). Interestingly, we revealed a significant upregulation of genes associated with senescence and SASP/inflammation in mutant oligodendrocytes (Supplemental Figure 7B, and Figure 8G), as also confirmed by qPCR analysis (Supplemental Figure 7C and Supplemental Figure 12, B–D).

As a complementary result, to confirm that the elevation of SASP/inflammation and ROS in mutant mice was due to senescent oligodendrocytes and not by activated microglia, we measured these molecules in PLX-treated *Cnp-Cre Jab1^fl/fl^* mice, thus ablated of microglia. SASP-related genes and ROS remained significantly elevated in mutant mice treated with PLX as compared with WT controls (treated and not treated; Supplemental Figure 8, A and B).

Collectively, loss of JAB1 induces progressive DNA damage in mutant oligodendrocytes that undergo a senescence-like program. Further, senescent oligodendrocytes trigger inflammation and promote diffuse oxidative stress leading to neurodegeneration.

### Jab1 deletion in adult mice induces neurodegeneration, neuroinflammation, and senescence.

**Figure 7:**
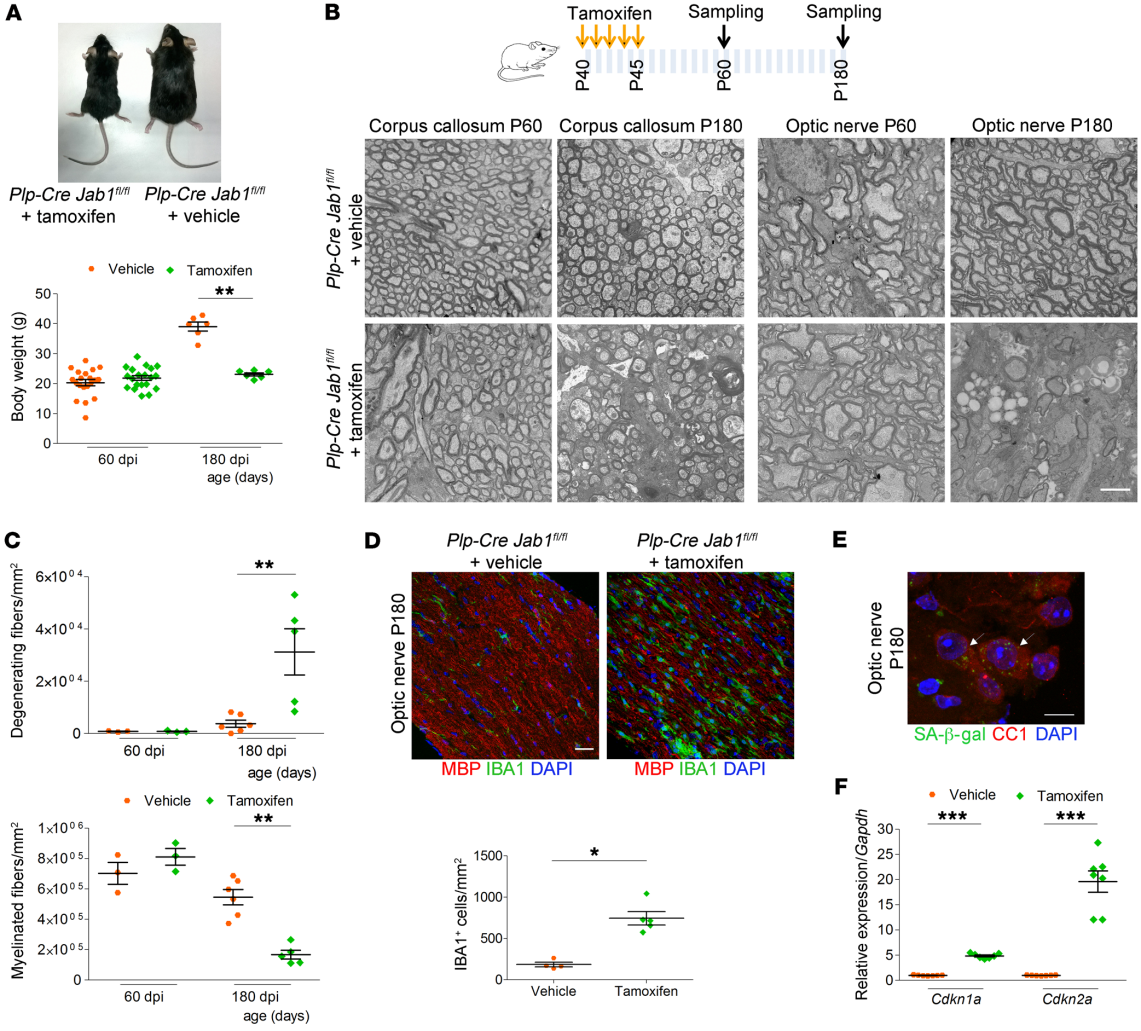
CNS demyelination, inflammation, and axonal degeneration are reproduced in *Plp-CreERT2 Jab1^fl/fl^* mice. (**A**) Tamoxifen-treated *Plp-CreERT2 Jab1^fl/fl^* mouse showing reduced size as compared with littermate control and quantification of body weight at P60 and P180 showing significant reduction at later time point (**P < 0.01; *n =* 18 at P60 and 6 at P180; 2-tailed nonparametric Mann-Whitney *U* test). dpi, days postinjection. (**B**) Schematic representation of tamoxifen administration and electron micrographs of P60 and P180 optic nerve and corpus callosum from tamoxifen-treated *Plp-CreERT2 Jab1^fl/fl^* and control (vehicle-treated *Plp-CreERT2 Jab1^fl/fl^*) mice, showing no differences at P60 but diffuse demyelination and axonal degeneration at P180. (**C**) Quantification showing significant increase in the number of degenerating fibers and decrease of myelinated fibers in P180 tamoxifen-treated *Plp-CreERT2 Jab1^fl/fl^* mice (**P < 0.01; *n =* 3 at P60 and 6 at P180; 2-tailed nonparametric Mann-Whitney *U* test). (**D**) Immunolabeling for IBA1 and MBP of P180 optic nerves from tamoxifen-treated *Plp-CreERT2 Jab1^fl/fl^* and control mice, and relative quantification showing significantly increased number of IBA1^+^ cells in tamoxifen-treated mice (*P < 0.05; *n =* 4 in controls and 5 in tamoxifen-treated mice; 2-tailed nonparametric Mann-Whitney *U* test). (**E**) β-Gal staining of the optic nerve from P180 tamoxifen-treated *Plp-CreERT2 Jab1^fl/fl^* and control mice double-stained with CC1, showing β-gal^+^ oligodendrocytes in tamoxifen-treated mice. (**F**) qPCR for *Cdkn1a* (p21^CIP1^) and *Cdkn2a* (p16^INK4a^), showing increased expression in the optic nerve from P180 tamoxifen-treated *Plp-CreERT2 Jab1^fl/fl^* as compared with control mice (***P < 0.001; *n =* 7; 2-tailed nonparametric Mann-Whitney *U* test). Scale bars: (**B**) 2 μm; (**D**) 40 μm; (**E**) 10 μm.

To further confirm the role of JAB1 in the regulation of the senescence program, we also deleted *Jab1* in mature oligodendrocytes using the inducible *Plp-CreERT2* transgene (30). *Plp-CreERT2 Jab1^fl/fl^* mice were treated with tamoxifen from P40 to P45. When they were bred with *EYFP-Rosa26* mice, PCR and Western blot analyses for JAB1 confirmed efficient recombination in optic nerves (Supplemental Figure 9, A–C). At 60 days after injection, tamoxifen-treated mice were phenotypically indistinguishable from controls in terms of behavior, weight, number of IBA1^+^ cells, CNS morphology (optic nerve, corpus callosum, and spinal cord), and myelination (Figure 7, A–C, and Supplemental Figure 9D). However, at 180 days after injection, tamoxifen-treated mice displayed reduced size and weight (Figure 7A). Morphological analyses showed diffuse CNS demyelination, loss of myelinated fibers, and axonal degeneration (Figure 7, B and C, and Supplemental Figure 9D). Further, these mice also showed an increase in IBA1^+^ microglia (Figure 7D). Similarly to *Cnp-Cre Jab1^fl/fl^* mice, tamoxifen-treated *Plp-CreERT2 Jab1^fl/fl^* mice had an increased number of oligodendrocytes (Supplemental Figure 9E) that expressed SA-β-gal (Figure 7E), and increased expression of p21^CIP1^, p16^INK4a^, and SASP (Figure 7F and Supplemental Figure 9F).

### Genetic depletion of p21^CIP1^ but not p16^INK4a^ ameliorates neurodegeneration in Jab1 mutants.

To test the hypothesis that senescence, through the p16^INK4a^ and/or p21^CIP1^ pathway, is responsible for neurodegeneration in *Jab1*-mutant mice, we ablated p16^INK4a^ or p21^CIP1^ in *Cnp-Cre Jab1^fl/fl^* mice.

Adult *Cnp-Cre Jab1^fl/fl^ p16^INK4a–/–^* mice did not show amelioration of demyelination and neurodegeneration of the CNS, as confirmed by quantification of myelinated fibers and *g*-ratio in the optic nerve (Supplemental Figure 10, A and B). Similarly, the inflammatory infiltrate did not change (Supplemental Figure 10C). Not surprisingly, double-mutant oligodendrocytes (with *Jab1* and *p16^INK4a^* deleted) showed senescence markers, including SA-β-gal activity and high p21^CIP1^ expression (Supplemental Figure 10, D and E), and SASP levels were unchanged in the CNS tissue in comparison with *Cnp-Cre Jab1^fl/fl^* mice (Supplemental Figure 10F).

**Figure 8:**
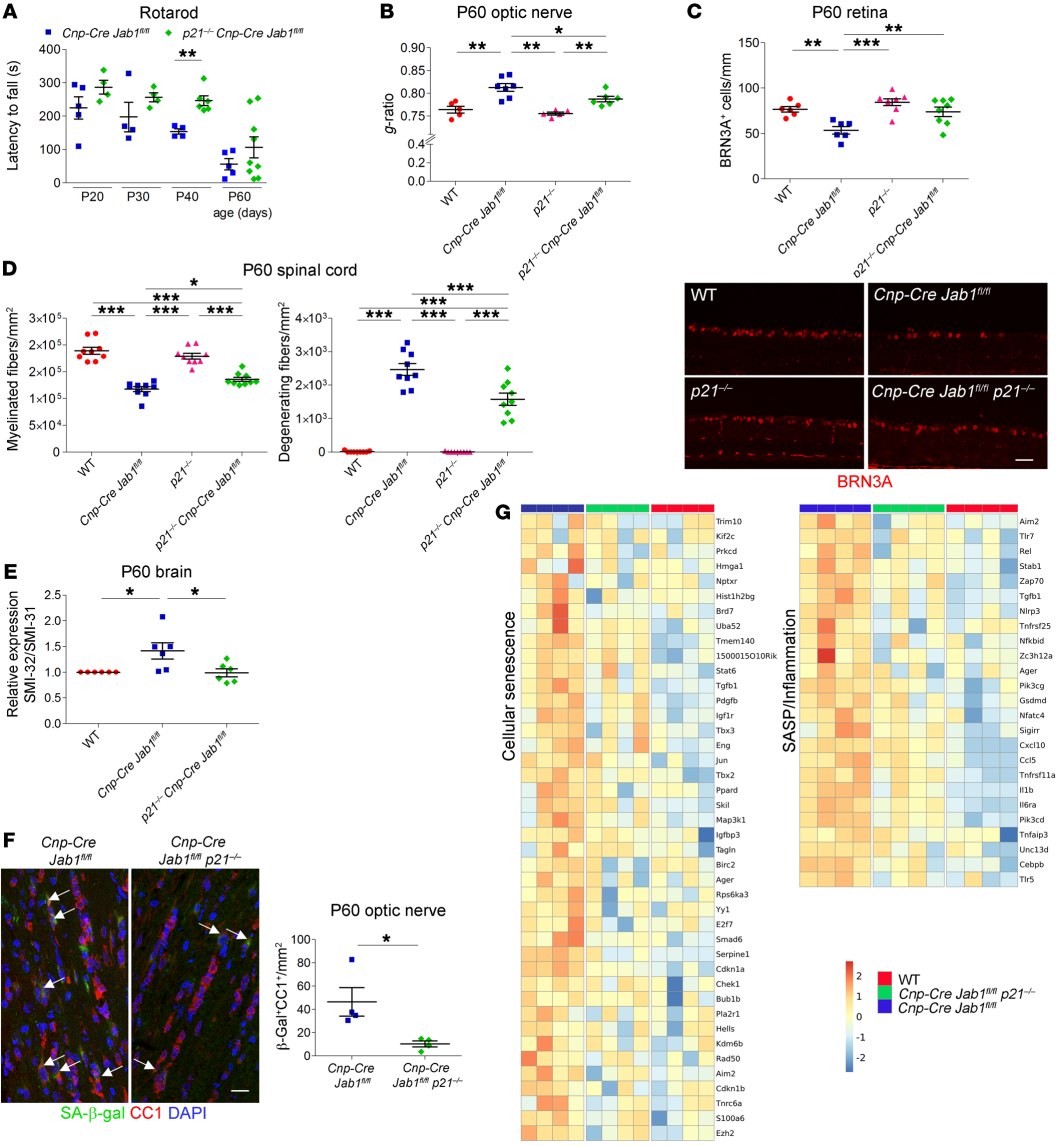
Deletion of *p21^CIP1^* ameliorates the phenotype in *Jab1*-mutant mice. (**A**) Rotarod analysis showing amelioration of motor deficits in *Cnp-Cre Jab1^fl/fl^ p21^CIP1–/–^* as compared with *Cnp-Cre Jab1^fl/fl^* mice (**P < 0.01 at P40; *n =* 5; 2-tailed nonparametric Mann-Whitney *U* test). (**B**) Quantification of *g*-ratio in P60 optic nerves, showing a significant reduction in *Cnp-Cre Jab1^fl/fl^ p21^CIP1–/–^* as compared with *Cnp-Cre Jab1^fl/fl^* mice (*P < 0.05, **P < 0.01; *n =* 5–7; at least 2000 fibers counted per mouse; 1-way ANOVA with Bonferroni’s multiple-comparison test). (**C**) Confocal images and quantification of BRN3A^+^ ganglion cells in the retina at P60, showing a significant rescue in *Cnp-Cre Jab1^fl/fl^ p21^CIP1–/–^* as compared with *Cnp-Cre Jab1^fl/fl^* mice (**P < 0.01, ***P < 0.001; *n =* 6–8; 1-way ANOVA with Bonferroni’s multiple-comparison test). (**D**) Quantification of myelinated fibers and degenerating fibers in the P60 spinal cord, showing significant rescue in *Cnp-Cre Jab1^fl/fl^ p21^CIP1–/–^* as compared with *Cnp-Cre Jab1^fl/fl^* mice (*P < 0.05, ***P < 0.001; *n =* 7–9; 1-way ANOVA with Bonferroni’s multiple-comparison test). (**E**) Quantification of Western blot analysis for phosphorylated (SMI-31) and non-phosphorylated (SMI-32) neurofilaments of high molecular weight in P60 brain homogenate, showing a significant rescue in *Cnp-Cre Jab1^fl/fl^ p21^CIP1–/–^* as compared with *Cnp-Cre Jab1^fl/fl^* mice (*P < 0.05; *n =* 6; 1-way ANOVA with Bonferroni’s multiple-comparison test). (**F**) Confocal immunolabeling for CC1 and β-gal staining and quantification in P60 optic nerve, showing reduced number of senescent oligodendrocytes in *Cnp-Cre Jab1^fl/fl^ p21^CIP1–/–^* as compared with *Cnp-Cre Jab1^fl/fl^* mice (*P < 0.05; *n =* 4; 2-tailed nonparametric Mann-Whitney *U* test). (**G**) Heatmaps representing the expression values of key genes for cellular senescence and for SASP/inflammation differentially expressed in FACS-sorted O1^+^ oligodendrocytes. Scale bars: (**C**) 40 μm; (**F**) 40 μm.

Conversely, deletion of p21^CIP1^ in *Cnp-Cre Jab1^fl/fl^* mice induced amelioration of the early phase of the phenotype and delay in neurodegeneration. Rotarod analysis showed better performances in *Cnp-Cre Jab1^fl/fl^ p21^CIP1–/–^* as compared with *Cnp-Cre Jab1^fl/fl^* mice, significantly at P40 (Figure 8A) — a time point of active neurodegeneration in *Jab1*-mutant mice (see also Figure 1H). Mouse body weight was similar between WT and *p21^CIP1–/–^* mice, and between *Cnp-Cre Jab1^fl/fl^* and *Cnp-Cre Jab1^fl/fl^ p21^CIP1–/–^* mice (Supplemental Figure 11A). When we analyzed optic nerves at P60, we confirmed amelioration of the histopathological findings, including significant increase of myelin thickness as sustained by *g*-ratio quantification (Figure 8B and Supplemental Figure 11, B and C). We then quantified the number of ganglion cells in the retina; this was significantly higher in *Cnp-Cre Jab1^fl/fl^ p21^CIP1–/–^* as compared with *Cnp-Cre Jab1^fl/fl^* mice and not different from controls (Figure 8C). Accordingly, the number of myelinated fibers in the spinal cord was also significantly increased in double mutants, with significantly reduced numbers of degenerating axons (Figure 8D). Similarly, the levels of non-phosphorylated neurofilaments in the brains of double-mutant mice were rescued (Figure 8E and Supplemental Figure 11D). Conversely, the number of microglia did not differ in double mutants as compared with *Cnp-Cre Jab1^fl/fl^* mice (Supplemental Figure 11E).

When we investigated senescent oligodendrocytes, we observed a significant reduction of SA-β-gal positivity in P60 optic nerves of *Cnp-Cre Jab1^fl/fl^ p21^CIP1–/–^* as compared with *Cnp-Cre Jab1^fl/fl^* mice (Figure 8F). Consistently, we also observed a significant reduction of cytoplasmic HMGB1 expression in double-mutant oligodendrocytes and reduced levels of SASP molecules (Supplemental Figure 11, F and H). The level of p16^INK4a^ (*Cdkn2a*) was unchanged in double mutants as compared with *Cnp-Cre Jab1^fl/fl^* mice (Supplemental Figure 11G), suggesting that it might partially compensate in the absence of p21^CIP1^.

Finally, we performed RNA-Seq analysis in FACS-sorted (O1^+^) oligodendrocytes to evaluate cell rescue of senescence and SASP/inflammatory markers. In parallel with findings in CNS tissue, we observed a rescue of senescence-associated and SASP/inflammatory molecules in *Cnp-Cre Jab1^fl/fl^ p21^CIP1–/–^* as compared with *Cnp-Cre Jab1^fl/fl^* oligodendrocytes, which had a pattern more similar to that of controls (Figure 8G and Supplemental Tables 2 and 3). Also, markers for oligodendrocyte maturation in double-mutant oligodendrocytes were more similar to controls as compared with *Cnp-Cre Jab1^fl/fl^* ones (Supplemental Figure 12A). As expected, qPCR for *p21^CIP1–/–^* (*Cdkn1a*) was undetectable in *Cnp-Cre Jab1^fl/fl^ p21^CIP1–/–^* oligodendrocytes (Supplemental Figure 12B). qPCR for selected markers of senescence and SASP (included in the heatmaps) confirmed RNA-Seq results (Supplemental Figure 12, C and D).

Overall, these above results suggest that interfering with the senescence pathway may ameliorate/delay neurodegeneration in *Jab1*-mutant mice.

### JAB1 expression in human brain.

**Figure 9:**
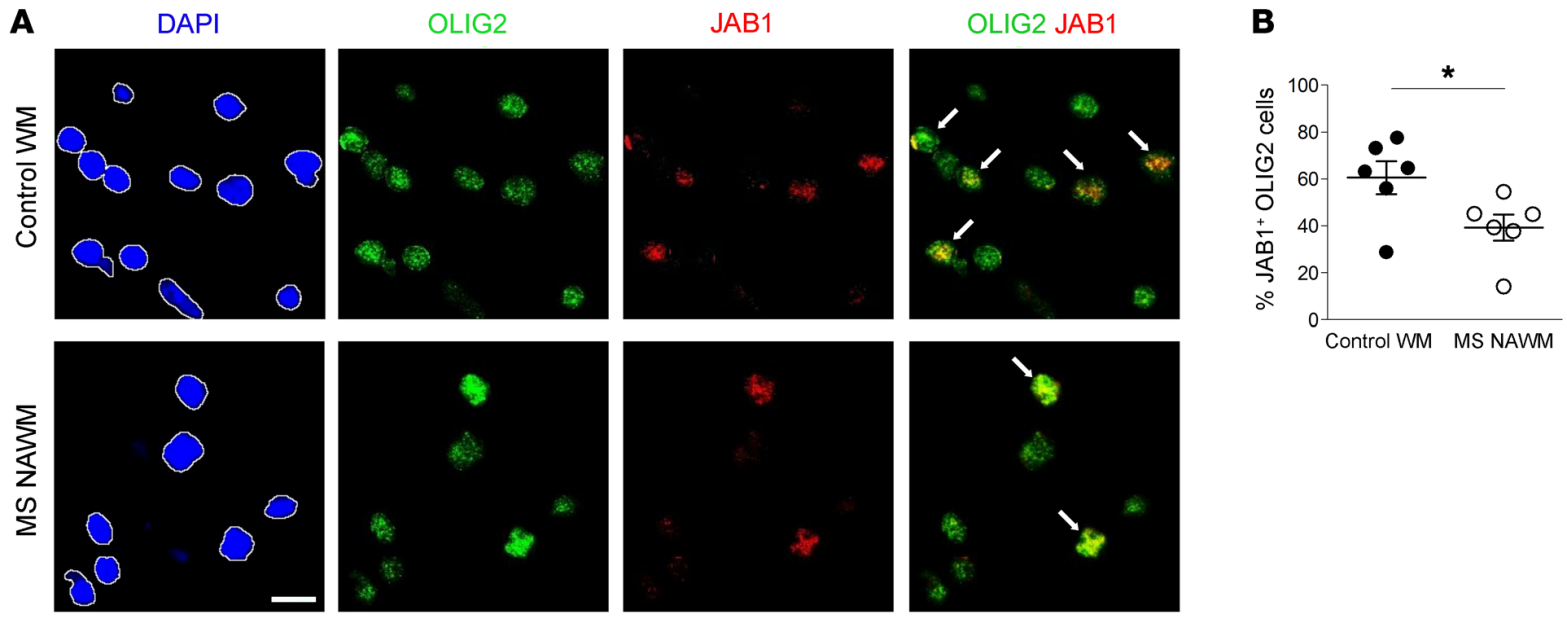
Expression of JAB1 in brain of MS patients. (**A**) Representative immunofluorescence images for nuclear JAB1 (red) expression in OLIG2^+^ cells (green) in control white matter (WM) or in normal-appearing white matter (NAWM) surrounding chronic inactive MS lesion. Regions of interest (ROIs) were generated on DAPI images (left panels) to select nuclei and then applied to the corresponding OLIG2 and JAB1 images. Arrows highlight JAB1^+^ oligodendrocytes. (**B**) Percentage of JAB1-expressing OLIG2^+^ cells in control white matter (black dots) or MS normal-appearing white matter (white dots). Each dot represents a single sample, and bars represent mean (*P < 0.05; *n =* 6; 2-tailed nonparametric Mann-Whitney *U* test). Scale bar: 10 μm.

As pathological findings in our mutant mice resemble those described in MS pathology, to strengthen the clinical relevance of our results we investigated JAB1 expression in brain samples from MS patients and controls. Immunofluorescence analysis revealed JAB1 expression in all the oligodendrocyte nuclei of normal control brain samples (Figure 9). Conversely, normal-appearing white matter surrounding chronic inactive MS lesions showed a significant reduction of oligodendrocytes that had JAB1^+^ nuclei (Figure 9, A and B).

## Discussion

Oligodendrocyte DNA damage and oxidative stress are among the main pathological mechanisms driving neurodegeneration in white matter disorders, such as progressive MS and leukodystrophies (8–11). However, the underlying molecular mechanisms have not been fully clarified, partly because mouse models do not completely recapitulate human disorders and/or pathogenesis (31, 32). We now report that genetic deletion of JAB1 in oligodendrocytes is sufficient to cause CNS demyelination, inflammation, and progressive neurodegeneration by inducing cell-autonomous DNA damage. Mutant oligodendrocytes undergo a senescence-like process that results in diffuse oxidative stress and proinflammatory cytokine release into the CNS. These events promote inflammation and neurodegeneration, leading to mouse death in few months. Moreover, our data strongly indicate that suppression of microglia activation does not ameliorate neurodegeneration. We provide a proof of principle that interfering with the senescence pathway ameliorates/delays the disease.

The role of JAB1 in oligodendrocytes has been corroborated using 2 different transgenes, confirming the cell-intrinsic role of oligodendrocytes in the pathogenesis of CNS demyelination, inflammation, and neurodegeneration. We posit a predominant role for postmitotic oligodendrocytes in causing these effects. Indeed, although recombination with the *Cnp-Cre* transgene has been reported also in NG2^+^ OPCs (30), and JAB1 has been previously associated with control of cell cycle progression and proliferation in Schwann cells (17), we did not observe any defect in OPC proliferation and differentiation. The different role of JAB1 in peripheral and central myelinating glia is additionally demonstrated by the fact that p27^KIP1^ levels did not change in oligodendrocytes, in contrast to Schwann cells (17), and conversely that Schwann cells did not elevate the senescence markers p16^INK4a^ and p21^CIP1^. Moreover, unlike Schwann cells that depend on JAB1 expression for survival (17), oligodendrocytes do not undergo apoptosis (at least until late stages and in low percentage). Indeed, oligodendrocytes survive despite extensive DNA damage, raising the possibility of their entry into a senescence-like program.

Cellular senescence is a programmed, transient phenomenon that normally contributes to tissue remodeling and homeostasis during development. In advanced age, senescent cells may accumulate following declining/disruption of the immune function, decreased stability of p53 (p21^CIP1^), and accumulation of DNA damage, which, however, does not reach a threshold level to induce cell death (33). Cellular senescence has been well investigated in mitotic cells and is mediated by the activation of either one or both p53/p21^CIP1^ and p16^INK4a^/pRB (retinoblastoma protein) tumor suppressor pathways (34). Both these pathways are complex, involve side branches, and are interlinked with extensive crosstalk. Activated p53 regulates the expression of many antiproliferative genes, primarily through p21^CIP1^. p21^CIP1^ arrests the cell cycle (by inhibition of various cyclin-dependent kinases [CDKs]), facilitates DNA damage response, induces the secretion of SASP, and promotes ROS elevation (35). p16^INK4a^ overexpression is also induced during senescence. p16^INK4a^ directly binds to CDK4/6, prevents the phosphorylation of pRB, and promotes the expression of EF2-target genes, acting as cell cycle brake. p16^INK4a^ also has non-canonical (pRB-independent) roles, regulating mitochondria biogenesis, oxidative stress, and inflammatory molecules (36). These chronic senescent cells promote or amplify cell/tissue damage through the release of inflammatory factors (SASP) and ROS (37). Based on the results of our study, we posit that *Jab1^–/–^* senescent oligodendrocytes couple CNS demyelination to chronic inflammation and elevated ROS production, which eventually result in axonal damage and loss. Accordingly, demyelination per se does not necessarily induce axonal degeneration, as reported in *shiverer* (*Mbp^–/–^*) mutants, which are devoid of CNS myelin but present preserved axons (7), indicating that further mechanisms may trigger neurodegeneration.

It is intriguing to note that senescent progenitors have been identified in brains of progressive MS patients as well as in induced pluripotent stem–derived neural precursor cells obtained from the same patients (38), providing evidence that cellular senescence is an active process during neurodegeneration. Notably, a reliable model reproducing key clinical and pathological features of primary progressive MS is an absolute requirement to validate possible therapeutic treatments for these patients (31), who are characterized by activated microglia, robust chronic demyelination, and autoimmune-independent neurodegeneration (31). These are all features that we observed in *Jab1*-mutant mice. In this context, oligodendrocyte-conditional *Jab1^–/–^* mice would promote cell-intrinsic DNA damage and defective DNA damage repair, which may represent a possible common mechanism of oligodendrocyte-driven neurodegeneration in different white matter disorders, such as progressive MS and leukodystrophies. Accordingly, we observed reduced JAB1 expression in oligodendrocytes of brain samples from MS patients. Moreover, oligodendrocyte DNA damage, senescence, oxidative stress, and defective DNA damage repair have all been described in progressive MS (8, 38–40) and leukodystrophy (41–44).

We consistently observed neuroinflammation in oligodendrocyte-conditional *Jab1^–/–^* mice as a consequence of primary oligodendrocyte dysfunction. Inflammation was primarily related to microglia activation, though lymphocytes, monocytes, and macrophages later contributed to the process. Predominant microglia activation is a common finding in both primary progressive MS and leukodystrophies (3, 39, 45, 46), and is observed in other models featuring oligodendrocyte dysfunction (10, 18). Given the phenotypic characteristics of our mutant, we could test whether microglia actively contributed to neurodegeneration. Interestingly, microglia depletion did not ameliorate demyelination nor neurodegeneration, suggesting that this is not a key event in disease progression. This result is in line with the failure in the use of immunomodulatory/immunosuppressive drugs to treat progressive MS patients (47). Our data indicate that cells other than inflammatory, likely senescent oligodendrocytes, with or without the contribution of reactive astrocytes, promote neurodegeneration. Nevertheless, we cannot exclude that enforcing neuroprotective microglia subtypes could constitute an alternative approach to rescue neurodegeneration in chronic white matter disorders (48).

In conclusion, we propose that oligodendrocyte senescence cell-autonomously represents a key element in the pathogenesis of neurodegeneration in chronic white matter disorders. Thus, targeting senescent cells might constitute a primary objective to develop innovative therapeutic strategies. Accordingly, our results show that interfering with the senescence pathway may ameliorate/delay the disease, as described also in other contexts (49, 50). Notably, this was observed with deletion of p21^CIP1^ but not p16^INK4a^. One explanation may reside in compensation. Not surprisingly, in both double mutants, the expression of the other senescence marker (p21^CIP1^ in *Cnp-Cre Jab1^fl/fl^ p16^INK4a–/–^* and p16^INK4a^ in *Cnp-Cre Jab1^fl/fl^ p21^CIP1–/–^* mice) was maintained at increased levels, and this may suggest that both these genes should be deleted to completely rescue the phenotype. Moreover, differences in the experimental rescue may be due to a specific function or pathway originated by the 2 cyclin inhibitors. For example, it is known that p21^CIP1^ is required (mainly) for the induction of senescence while p16^INK4a^ is required for its maintenance (34). Epigenetically induced senescence mostly acts through p16^INK4a^, as opposed to DNA damage–induced senescence, which mainly relies on p21^CIP1^ (51). Moreover, specific functions of p21^CIP1^ and p16^INK4a^ in postmitotic cells, such as oligodendrocytes, are still poorly understood. We cannot exclude that senescence in CNS cells (such as oligodendrocytes) might be sustained also by other molecules that may partially take over the function of p21^CIP1^, and/or may not include p16^INK4a^.

Unfortunately, senolytic drugs still present limits in terms of potency and applicability (52, 53). However, recent work gave promising results by eliminating senescent cells with antibodies or chimeric antigen receptor T cells (54, 55), suggesting the possibility of developing alternative treatments for these devastating disorders of the CNS.

## Methods

### Animals.

The generation of floxed *Jab1*, *Cnp-Cre*, *Plp-CreERT2*, and *EYFP-Rosa26* mice has been previously described (18, 30, 56, 57). *Ccr2* and p21^CIP1^ (*Cdkn1a*) knockout mice were purchased from The Jackson Laboratory, and p16^INK4a^ (*Cdkn2a*) knockout mice from the National Cancer Institute Mouse Models of Human Cancers Consortium (Supplemental Table 4). For genotyping, we isolated genomic DNA from tail biopsies using DirectPCR solution (ViaGen), according to the manufacturer’s directions. All primer sequences are available in Supplemental Table 5.

### PLX3397 administration.

The compound pexidartinib (PLX3397; S7818) was purchased from Selleck Chemicals and formulated in irradiated D10001i Rodent Diet standard chow by Research Diets at a dose of 290 mg per kilogram of chow. At P20 WT and *Cnp-Cre Jab1^fl/fl^* mice were fed with standard diet or PLX3397 (290 mg/kg) chow for 21 days to reduce microglia cells by more than 99% in the CNS, as previously described (25).

### Behavioral test: rotarod.

Mice at different ages were placed on a 3-cm-diameter horizontal rotating rod with acceleration from 4 rpm to a maximum of 40 rpm after 300 seconds (4 rpm every 30 seconds). The maximum time on the rod was 600 seconds. The latency to fall was measured on the moment the mouse fell down or stopped walking (clung to the rod) for 2 consecutive turns. Mice were trained the first day with a constant speed of 20 rpm for 5 minutes. On days 2 and 3, mice were directly subjected to 3 trials in the morning and 3 trials in the afternoon (each trial was separated at least by 15 minutes).

### MRI.

For each scan session, mice were anesthetized using gas anesthesia (1%–2% isoflurane in O_2_) with the respiration rate and external body temperature monitored during imaging using a magnetic resonance–compatible small-animal monitoring and gating system. External body temperature was maintained at 36°C–37°C with a heating circulator bath. With a dedicated phased-array surface coil placed on the heads of the mice, MRI acquisitions were performed on a 7-Tesla scanner (70/30 cm, Bruker BioSpin MRI GmbH) using different magnetic resonance sequences. Following standard anatomical images (T2 weighted) in the 3 orthogonal planes, 16 coronal sections (0.9 mm thickness) were positioned to cover the entire mouse brains. Magnetization transfer (MT) data were acquired using a fast low-angle shot (FLASH) sequence (repetition time [TR]/echo time [TE] = 550/2.9 ms; 6 averages; matrix = 192 × 128) with a saturation pulse (frequency offset = 3000 Hz; radio frequency [RF] power = 5 microteslas) and with a high spatial resolution (75.5 × 70.3 μm^2^). The percentage of MT was quantified from the difference of signal in images with and without saturation pulse. Diffusion tensor imaging (DTI) was acquired with an echo planar imaging sequence (TR/TE = 4500/23 ms; matrix = 128 × 128) with diffusion gradients applied in 30 directions (*b* = 1200 s/mm^2^ and duration/separation *=* 5/10 ms) or without (*n =* 5, *b* = 0). From DTI, the main diffusion directions and the magnitude of diffusivity were calculated in all 3 directions using standard algorithms (Bruker-Paravision 5.1) that provide maps of the fractional anisotropy (FA), mean diffusivity (MD), axial diffusivity (λ_||_), and radial diffusivity (λ_⊥_). The total imaging session was approximately 1 hour. For each animal scan, MRI parameters were measured by manual drawing of regions of interest (ROIs) in the medial corpus callosum over different coronal sections. An FA map was used for definition of ROI limits, as the corpus callosum was clearly visible. All measured values were normalized to the mean value of healthy controls (100%).

### VEP recording.

Noninvasive epidermal VEPs were recorded using a 6 mm Ø Ag/AgCl cup electrode (SEI EMG s.r.l.) placed on the shaved scalp over V1 (1 mm anterior to interaural line and 2.5 mm contralateral to stimulation) fixed with electroconductive adhesive paste (Elefix EEG paste). A needle was inserted on the nose as reference. Mice were anesthetized (80 mg/kg ketamine, 10 mg/kg xylazine) at adequate level verified as absence of tail-pinching reflex. Body temperature was maintained at 36.5°C ± 0.5°C by a homeothermic blanket system with a rectal thermometer probe. Pupils were dilated with 1% tropicamide (Visumidriatic, Visufarma s.p.a.) and eyes protected with ophthalmic gel (2% hydroxypropylmethylcellulose; Gel 4000, Bruschettini). VEPs were acquired after 5 minutes of dark adaptation in a darkened Faraday cage. For each VEP recording session, 3 trains of 20 flash stimuli (with intensity 260 mJ, 10 microseconds duration, and 1 Hz frequency) were delivered with a flash photostimulator (Micromed) placed at 15 cm from the stimulated eye, while the contralateral was covered. Data were acquired by Micromed SystemPlus Evolution software at a sampling frequency of 4096 Hz (bandpass-filtered 0.16–1024), coded with 16 bits, and bandpass-filtered (5–100 Hz, notch filter 50 Hz). When no VEP response was identified (signal lower than 5 μV), an additional recording was performed, keeping the flash outside the Faraday cage to ensure noise reduction below that level. Latency of the first negative peak (N1) and amplitude with respect to the following positive peak (P2) were measured with the same software.

### Histology and morphometric analysis.

Semithin and ultrathin morphological experiments were performed as previously described (58). Briefly, brain, spinal cord, and optic nerves were dissected from mice transcardially perfused with 4% (vol/vol) paraformaldehyde, fixed with 2% (vol/vol) glutaraldehyde and 4% (vol/vol) paraformaldehyde in 0.12 M phosphate buffer overnight, postfixed with 1% (vol/vol) osmium tetroxide, and embedded in Epon (Fluka). Semithin sections (0.5 μm thick) were stained with toluidine blue and examined by light microscopy (Olympus BX51, Leica Microsystems). Ultrathin sections (100–120 nm thick) were stained with uranyl acetate and lead citrate and examined by electron microscopy (FEI Talos L120C G2 Transmission Electron Microscope). Morphometric analysis on ultrastructural sections of optic nerve was done on 25 digitized images per animal, randomly photographed at ×5300, whereas for spinal cord we analyzed images of semithin sections at ×100, 5 images per mouse. *G*-ratio values were determined by manual measurement of axon and fiber diameters, while fiber count was defined by absolute number of fibers per area (mm^2^) with ImageJ software (version 1.6, NIH; http://rsbweb.nih.gov/ij/download).

### Immunohistochemistry.

Mice were anesthetized and perfused with 4% (vol/vol) paraformaldehyde. Brain, spinal cord, and optic nerves were isolated and postfixed overnight in 4% (vol/vol) paraformaldehyde and incubated before cryoprotection in 30% sucrose/PBS at 4°C overnight. Tissue was embedded in OCT compound and sectioned. Eight-micrometer-thick cryosections were permeabilized in cold acetone or methanol depending on the antibodies used. Sections were incubated in blocking solution containing 10% Normal Goat Serum (Vector Laboratories) in BSA 1% (Sigma-Aldrich) with 1% Triton X-100 (Sigma-Aldrich) if required. Primary antibodies (Supplemental Table 6) were diluted in blocking solution containing 1% Triton X-100, if required, while secondary antibodies (Supplemental Table 7) were diluted in phosphate buffer. Slices were then mounted with Vectashield with DAPI (H-1200, Vector Laboratories). For immunoperoxidase staining, sections were processed with UltraVision Quanto Detection System HRP-DAB (Thermo Fisher Scientific) following the manufacturer’s instructions. Briefly, samples were incubated for 10 minutes with UltraVision Hydrogen Peroxidase Block, washed 2 times with TBS-T (0.05% Tween-20, Sigma-Aldrich), and incubated for 5 minutes with UltraVision Protein Block and successively with primary antibody for 1 hour at room temperature. After washing, sections were incubated for 10 minutes with Primary Antibody Amplifier Quanto, and then with HRP Polymer Quanto for a further 10 minutes (Thermo Fisher Scientific). Staining was revealed by DAB Quanto Chromogen (5 minutes) to detect HRP (Thermo Fisher Scientific). Slides were examined with confocal (Leica SP5 or SP2, Leica Microsystems), fluorescent, or light microscope (Olympus BX51, Leica Microsystems). Quantification analysis was performed by counting of absolute number of positive cells per area (mm^2^) in 5 adjacent images per tissue section, in 2–3 serial sections.

### Retina isolation and immunofluorescence of retinal ganglion cells.

Mice were anesthetized and perfused with 4% (vol/vol) paraformaldehyde. The eyes were dissected out and postfixed with 4% (vol/vol) paraformaldehyde for 60 minutes. Retinas were isolated under a dissecting microscope. Briefly, after a circumferential cut of the cornea was made following the limbus, the cornea and lens were removed, and the retina was visualized as a white surface covering the inside of the posterior eye cup. Eye cups were equilibrated in 30% sucrose/PBS overnight, embedded in OCT compound, and frozen in liquid nitrogen. Ten-micrometer-thick cryosections of retina were processed for immunofluorescence as described above. To count retinal ganglion cells (RGCs), 4 non-overlapping images from 3 different retinal sections were taken at the fluorescence microscope (Olympus BX5, Leica Microsystems) using a ×20 objective. RGCs were manually counted as a ratio of RGCs per millimeter, and the average value was calculated for each sample.

### BrdU assays.

Optic nerves were evaluated in vivo by BrdU incorporation (5-bromo-2′-deoxyuridine; Roche), as previously described (17). To evaluate OPC differentiation into mature CC1^+^ oligodendrocytes, we injected BrdU (i.p., 100 mg/g body weight) in P20 mice for 3 consecutive days and analyzed mice at P30. Briefly, cryosections were fixed in cold methanol, treated with 2N HCl for 15 minutes at 37°C, and neutralized with 0.1 M sodium borate (pH 8.5) for 10 minutes. Slides were then incubated overnight with anti-NG2 polyclonal antibody or anti-CC1 mAb and anti-BrdU mAb to identify proliferating cells at P20 and differentiated oligodendrocytes at P30. After staining with secondary antibodies, nuclei were counterstained with DAPI (Vectashield, Vector Laboratories). Quantification was performed by counting of the absolute number of positive cells per area (mm^2^) in 5 adjacent images per nerve section, in 2–3 serial sections (at least 4000 cells per animal).

### Senescence β-gal staining.

Mice were anesthetized and perfused with 4% (vol/vol) paraformaldehyde. Optic nerves were isolated and postfixed overnight in 4% (vol/vol) paraformaldehyde and incubated before cryoprotection in 30% sucrose/PBS at 4°C overnight. Tissue was embedded in OCT compound and sectioned. Eight-micrometer-thick cryosections were processed with CellEvent Senescence Green Detection Kit (Invitrogen) or Senescence Cell Histochemical Staining Kit (Sigma-Aldrich) according to the manufacturers’ instructions. Briefly, optic nerve cryosections were postfixed for 15 minutes in 4% (vol/vol) paraformaldehyde, then washed and incubated at 37°C in a humid chamber for 2 hours with probe diluted into the prewarmed staining mixture. Finally, slides were processed for immunohistochemistry as previously described.

### ROS detection by electron paramagnetic resonance.

ROS were measured by electron paramagnetic resonance (EPR) spectroscopy, as previously described (59, 60). Briefly, tissue samples (optic nerve, spinal cord, and brain) were dissected and immediately incubated at 37°C in Krebs-HEPES buffer (KHB) containing 25 μM deferoxamine methane-sulfonate salt (DF) chelating agent and 5 μM sodium diethyldithio-carbamate trihydrate (DETC) at pH 7.4 with 1 mM 1-hydroxy-3-methoxycarbonyl-2,2,5,5-tetramethylpyrrolidine (CMH; Noxygen Science Transfer & Diagnostics) as spin probe. After 30 minutes the isolated tissues were placed in the center of a 1 mL plastic syringe according to Dikalov et al. (60), snap-frozen, and stored at –80°C. Then the frozen block was removed by gentle pushing from the warmed-up syringe and analyzed in the quartz Dewar with liquid N_2_. Spectra were recorded at 77 K using X-band (9 GHz) EPR spectroscopy (E-scan, Bruker BioSpin GmbH). The EPR signal, generated by the reaction of the spin probe (CMH) with whole-tissue ROS, was then recorded (acquisition parameters: modulation amplitude, 5 G; centered field, 2.0023 g; sweep time 10 seconds; field sweep, 60 G; microwave power, 1 mW; number of scans, 10; receiver gain, 1 × 10^3^**)**. Spectra were recorded and analyzed using Win EPR software (2.11 version) standardly supplied by Bruker. EPR measurements allowed us to attain a relative quantitative determination of ROS production rate in samples. All data were, in turn, converted into absolute concentration levels (micromoles per gram) by adopting CP^•^ (3-carboxy-2,2,5,5-tetramethyl-1-pyrrolidinyloxy) stable radical as external reference.

### Western blotting.

Proteins were isolated from snap-frozen tissue and homogenized in lysis buffer containing 2% (vol/vol) SDS, 25 mM Tris-HCl (pH 7.5), 95 mM NaCl, 10 mM EDTA (pH 8), phosphatase inhibitor (PhoSTOP, Roche), and protease inhibitors (cOmplete, Mini, EDTA-free, Roche). Homogenates were sonicated and centrifuged for 10 minutes at 15,294*g* at 4°C. Protein concentrations were determined by BCA Assay (Pierce). Equal amounts of homogenates were fractionated by SDS-PAGE and blotted onto PVDF (Millipore) or nitrocellulose (Bio-Rad). Membranes were blocked in 5% no-fat dry milk in PBS-T (0.1% Tween-20, Sigma-Aldrich, in PBS), incubated with specific primary and secondary antibodies (Supplemental Tables 6 and 7), washed in PBS-T, and developed with the Clarity Western ECL substrate (Bio-Rad) on films or analyzed by ChemiDoc Imaging System (Bio-Rad) or using the Odyssey Infrared Imaging System (LI-COR Biosciences) according to the manufacturer’s instructions. Densitometric analyses were performed with the ImageJ software for membranes developed by chemiluminescence and the Odyssey v3 CLX Infrared Imaging System when fluorescent secondary antibody was used. Each antigen was normalized with respect to the housekeeping protein.

### FACS analysis and cell sorting.

Mice were anesthetized and perfused with saline and the brain removed. Brains from 8 WT and 8 *Cnp-Cre Jab1^fl/fl^* mice at P40 and P60 were dissociated with 0.4 mg/mL of collagenase type IV (Sigma-Aldrich) and through an 18G syringe to obtain a homogeneous cell suspension. Cell suspensions were further enriched by an isotonic Percoll gradient, myelin removed, and cells washed in PBS and finally centrifuged and resuspended in MACS buffer as previously described (61). Cells were stained with specific antibodies (Supplemental Table 6). Zombie NIR (BioLegend) was added to exclude nonviable cells. Samples were sorted on FACS Aria Fusion (BD Biosciences) and analyzed with FlowJo (Tree Star). Schematic representation of gating strategy for FACS is depicted in Supplemental Figure 1. Mature oligodendrocytes were identified as single live cells CD45^–^O1^+^, activated microglia as single live CD45^+/lo^CD11b^+^Ly6G^–^Ly6C^–^CD11c^+^ cells, macrophages as single live CD45^hi^CD11b^+^Ly6G^–^Ly6C^–^ cells, monocytes as single live CD45^+^CD11b^+^Ly6G^–^Ly6C^+^ cells, lymphocytes as single live CD45^+^CD11b^–^ cells, and neutrophils as single live CD45^+^CD11b^+^Ly6G^+^ cells. In FACS sorting experiments, reanalysis by cytometry did not show overt CD45^+^ cell contamination (0% of CD45^+^ cells) in all the groups tested (WT, *Cnp-Cre Jab1^fl/fl^,* and *Cnp-Cre Jab1^fl/fl^ p21^CIP1–/–^*). Purity of sorted O1^+^ cells ranged between 80% and 90% on reanalysis. Owing to the FACS technique, to the material tested (CNS tissue after dissociation), and to the cells studied (CNS oligodendrocytes), a small contamination by other cells cannot be entirely excluded.

### Tissue irradiation.

Mice were anesthetized and perfused with saline, and optic nerves were dissected and placed in 500 μL of DMEM supplemented with 5% FBS (FBS Good, PAN Biotech) on ice in a 4-well plate. Optic nerves were cut to obtain 5-mm segments, and then segments were irradiated (3 Gy) by a BIOBEAM GM 8000 gamma irradiator (Gamma-Service Medical GmbH). Optic nerves were maintained in a cell incubator at 37°C, 5% CO_2_, for 8 hours and gently shaken every 2 hours. After 8 hours, optic nerves were embedded in OCT compound and snap-frozen in liquid nitrogen. Longitudinal 8-μm-thick cryosections were processed for immunohistochemistry as described above.

### Quantitative reverse transcriptase PCR.

Total RNA was isolated from optic nerves or sorted oligodendrocytes using TriPure Isolation Reagent (Roche) according to the manufacturer’s instructions. Briefly, cells or nerves were homogenized in the presence of TriPure Isolation Reagent, and total RNA was extracted with chloroform and precipitated with isopropanol. A portion (350 ng) of optic nerve total RNA was reverse-transcribed using High-Capacity cDNA Reverse Transcription Kit (Applied Biosystems). RNA from sorted oligodendrocytes was amplified and reverse-transcribed by SMART-Seq v4 Ultra Low Input RNA Kit (Takara Bio USA), and 1 μL of the amplified cDNA was validated using the Agilent 2100 Bioanalyzer and Agilent’s High Sensitivity DNA Kit (catalog 5067-4626). Quantitative reverse transcriptase PCR analyses were performed on Applied Biosystems 7900HT Real-Time PCR System using the 2× TaqMan PCR Master Mix (Applied Biosystems) according to the manufacturer’s recommendations. The primers used were TaqMan Gene Expression Assays by Applied Biosystems: *Ccl5* ID, Mm01302427-m1; *Cdkn1a* ID, Mm04205640-g1; *Cdkn2a* ID, Mm00494449-m; *Cops5* ID, Mm00489065-m1; *Csf2* ID, Mm01290062-m1; *Cxcl1* ID, Mm04207460-m1; *Gapdh* ID, Mm99999915-g1; *Il1b* ID, Mm00434228-m1; *Serpine1* ID, Mm00435858-m1; *Tgfb1* ID, Mm03024053-m1; *Trim10* ID, Mm00600002-m1; Trp53 ID, Mm01731287-m1. Levels of gene expression were determined with the comparative cycle threshold (ΔΔCt) method. The mRNA level of each gene of interest was normalized to the level of *Gapdh* mRNA. Each time point is the average of 4–5 experiments (each experiment was performed with 2 optic nerves, 1 brain hemisphere, or sorted oligodendrocytes derived from 2 mouse brains).

### RNA sequencing.

Total RNA was isolated from sorted oligodendrocytes using ReliaPrep RNA Cell Miniprep System (Promega) according to the manufacturer’s instructions. Four samples per genotype of WT, *Cnp-Cre Jab1^fl/fl^*, and *Cnp-Cre Jab1^fl/fl^ p21^CIP1–/–^* (1 sample derived from 2 different P40 mouse brains) were analyzed. Libraries were prepared using the SMART-Seq v4 Ultra Low Input RNA Kit for Sequencing (Takara Bio USA) according to the manufacturer’s instructions. Sequencing was performed on an Illumina NovaSeq 6000 machine, obtaining an average of 100 million paired-end reads per sample. The raw reads produced from sequencing were trimmed using Trimmomatic, version 0.32, to remove adapters and to exclude low-quality reads from the analysis. The remaining reads were then aligned to the murine genome GRCm38 using STAR, version 2.5.3a. Reads were eventually assigned to the corresponding genomic features using featureCounts (https://rnnh.github.io/bioinfo-notebook/docs/featureCounts.html), according to the GENCODE basic annotations (GENCODE version M22). Quality of sequencing and alignment was assessed using the FastQC (https://www.bioinformatics.babraham.ac.uk/projects/fastqc/), RseQC (http://rseqc.sourceforge.net/), and MultiQC (https://multiqc.info/) tools. Expressed genes were defined as those genes showing at least 1 count per million reads on at least 4 samples, corresponding to the sample size of each group of samples (62). Low-expressed genes that did not match this criterion were excluded from the data set. Gene expression read counts were exported and analyzed in the R environment (v3.5.1) to identify differentially expressed genes, using the DESeq2 Bioconductor library (v1.22.1; https://bioconductor.org/packages/release/bioc/html/DESeq2.html). The *P* values were adjusted using a threshold for false discovery rate (FDR) less than 0.05 (63). Functional enrichment analysis was conducted using the Enrichr R package (v1.0; https://doi.org/10.1093/nar/gkw377), starting from the lists of differentially expressed genes as defined by FDR < 0.05. The enrichment with a subset of selected gene sets was further evaluated using preranked gene set enrichment analysis (GSEA), as previously reported (64). The gene sets included in the GSEA analyses were retrieved from the Gene Ontology Biological Process collection as reported in the MSigDB database, and from the senescence pathway as previously described (65).

### Human brain samples.

Postmortem brain white matter samples of 6 multiple sclerosis (MS) chronic inactive plaques (1 female and 5 male, mean age 64 ± 10) and 6 normal control tissue donors (2 female and 4 male, mean age 60 ± 20) were conserved as snap-frozen tissue blocks at the NeuroResource tissue bank, UCL Queen Square Institute of Neurology. For lesion classification, tissue sections were analyzed by immunohistochemistry/immunofluorescence for myelin basic protein (MBP), CD68 (microglia/macrophages), GFAP (astrocytes), and CD3 (T cells), as previously reported (66, 67). The chronic inactive lesions were fully demyelinated, lacked T cell infiltration in the parenchyma, and presented sharp lesion borders, strong astrogliosis, and low myeloid cell numbers. These human samples were donated with informed consent using documentation approved by the National Health Service (National Research Ethics Service Committee London–Central, UK), and stored under a Research Sector License from the UK Human Tissue Authority.

### Human brain sample immunostaining.

Twelve-micrometer-thick serial sections were prepared from human snap-frozen brain tissue blocks. Sections were fixed in 4% (vol/vol) paraformaldehyde, and nonspecific binding was blocked in PBS/1% BSA/5% FCS. Slides were then incubated with primary antibodies and appropriate species-specific Alexa Fluor 488/594–conjugated secondary antibodies (Thermo Fisher Scientific). The slides were counterstained with DAPI (Sigma-Aldrich) and mounted with fluorescent mounting medium (Agilent). For primary antibodies, see Supplemental Table 6. As negative controls, mouse IgG1 (Sigma-Aldrich) or rabbit immunoglobulins (Thermo Fisher Scientific) were used at the same concentration as the primary antibodies. Fluorescence images were captured with a Leica TCS SP5 confocal laser-scanning microscope equipped with ×40 and ×63 oil objectives (Leica Microsystems) for tissue stainings. Single stacks were acquired for image quantification. LAS AF software (Leica Microsystems) was used for image acquisition, and ImageJ (v1.6.0) software was used for image analysis. To quantify nuclear JAB1, DAPI images were converted to 8-bit, and regions of interest (ROIs) were generated to select (DAPI^+^) nuclei. Then ROIs were applied to the corresponding OLIG2 or JAB1 images, and the number of positive nuclei was counted after setting of appropriate fluorescence threshold.

The data discussed in this publication were deposited in the NCBI’s Gene Expression Omnibus database (GEO GSE184573).

### Statistics.

Data were analyzed by log-rank (Mantel-Cox) test for Kaplan-Maier curve, 2-way ANOVA with Bonferroni’s post hoc correction for longitudinal study, and unpaired *t* test or 2-tailed nonparametric Mann-Whitney *U* test to compare 2 groups at the same time point. Normalized data were analyzed using 1-sample 2-tailed Student’s *t* test. From data sets from more than 2 groups, dedicated *P* values were corrected for multiple testing using 1-way ANOVA with Bonferroni’s multiple-comparison test. All these tests were done using GraphPad Prism, version 5. Differences were considered significant when *P* was less than 0.05. Data are presented as mean ± SEM, and levels of significance are depicted by asterisks in the figures: *P < 0.05, **P < 0.01, ***P < 0.001, ****P < 0.0001.

### Study approval.

All animal experiments were approved by and performed in compliance with the guidelines of the San Raffaele Scientific Institute Institutional Animal Care and Use Committee. Human studies were approved by the Ethical Committee at San Raffaele Scientific Institute and performed in accordance with the ethical standards laid down in the 1964 Declaration of Helsinki and its later amendments or comparable ethical standards.

## Author contributions

SCP designed and supervised the study. CR, GD, and EC performed histological and morphological analyses. CR performed immunohistochemistry, β-gal, and Western blot analyses. CR, MB, and GSG performed the FACS analysis. CR and EP performed reverse transcriptase and quantitative PCR experiments. LL and SM performed VEP analysis. LC performed MRI studies. SMS and AV performed ROS quantification. CF, EC, and JN performed studies in human samples. SCP, CR, AQ, MB, KAN, RP, and CF interpreted the results. SCP wrote the paper with input from all the authors. SCP, CR, AQ, and CF made the figures.

## Supplementary Material

Supplemental data

Supplemental Table 1

Supplemental Table 2

Supplemental Table 3

## Version 1 12/07/2021

In-Press Preview

## Version 2 02/01/2022

Electronic publication
