# Supplementary material for: JAB1 deletion in oligodendrocytes causes senescence-induced inflammation and neurodegeneration in mice
Source: J Clin Invest. 2022 Feb 1;132(3):e145071. doi: 10.1172/JCI145071 (PMC8803330; doi:10.1172/JCI145071)
Supplement: Supplemental data [file jci-132-145071-s251.pdf]

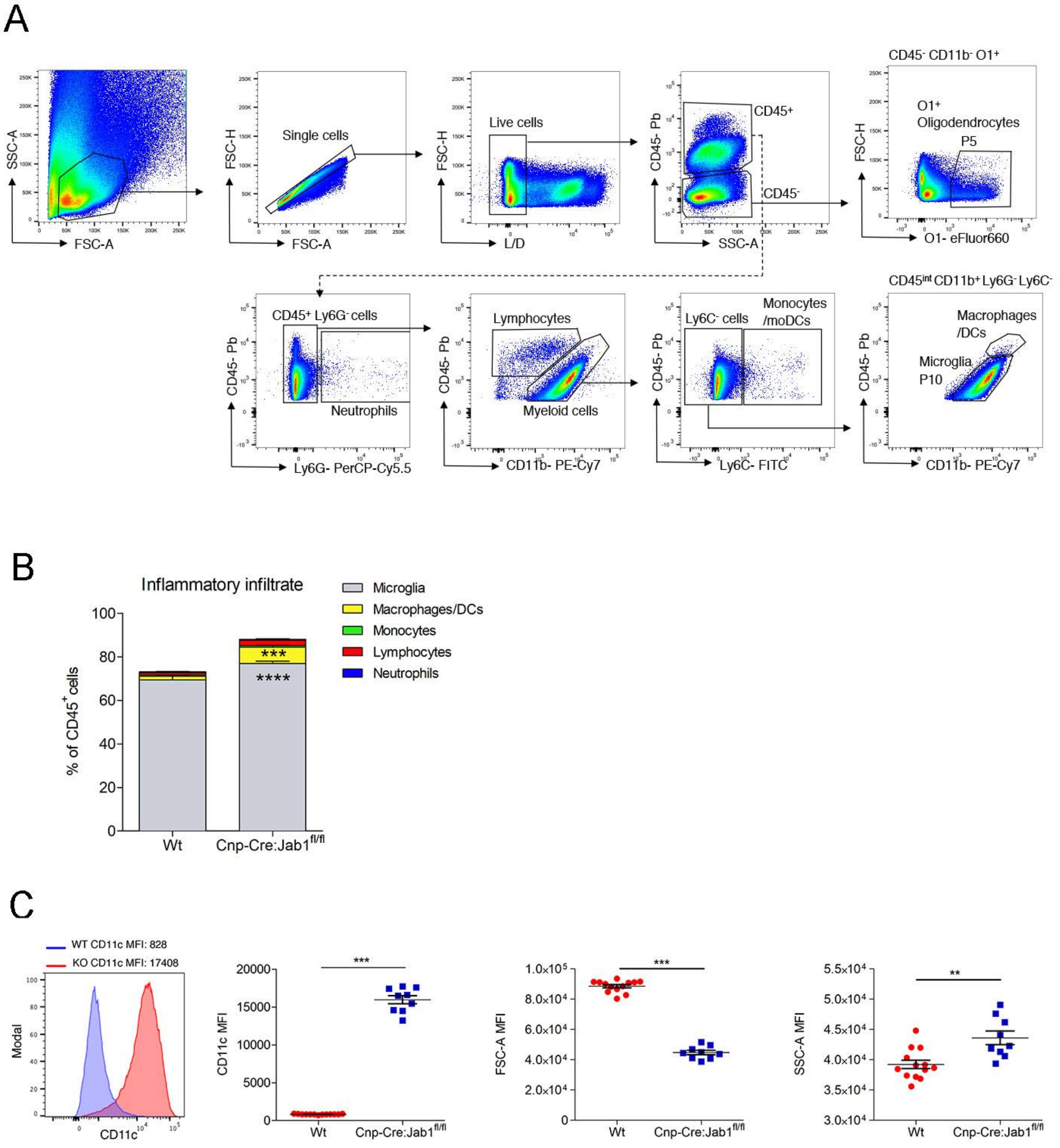

Supplemental Figure 1. FACS analysis of inflammatory cells in mutant mice.
(**A**) Schematic representation of gating strategy for FACS-sorting of O1+ oligodendrocytes (P5, CD45$^-$ CD11b$^-$ O1$^+$ cells) and microglia (P10, CD45int CD11b$^+$ Ly6G$^-$ Ly6C$^-$ cells) from Wt, *Cnp-Cre:Jab1$^{fl/fl}$* and *Cnp-Cre:Jab1$^{fl/fl}$;p21CIP1$^{-/-}$* mice at P40. (**B**) Representation of the inflammatory infiltrate at P60 in the brain of *Wt* and *Cnp-Cre:Jab1$^{fl/fl}$* mice. ***p<0,001, ****p<0,0001 (n=13 for Wt, n=9 for Cnp-Cre:Jab1$^{fl/fl}$; Two-way ANOVA with Bonferroni post-correction). (**C**) Quantification of the activated microglia (CD11c), and morphological parameters (FSC, Forward scatter: smaller means more active; SSC, side scatter: increased grainy means more active; **p<0,01, ***p<0,001; n=13 for Wt, n=9 for *Cnp-Cre:Jab1$^{fl/fl}$*; Two-tailed nonparametric Mann–Whitney U-test).

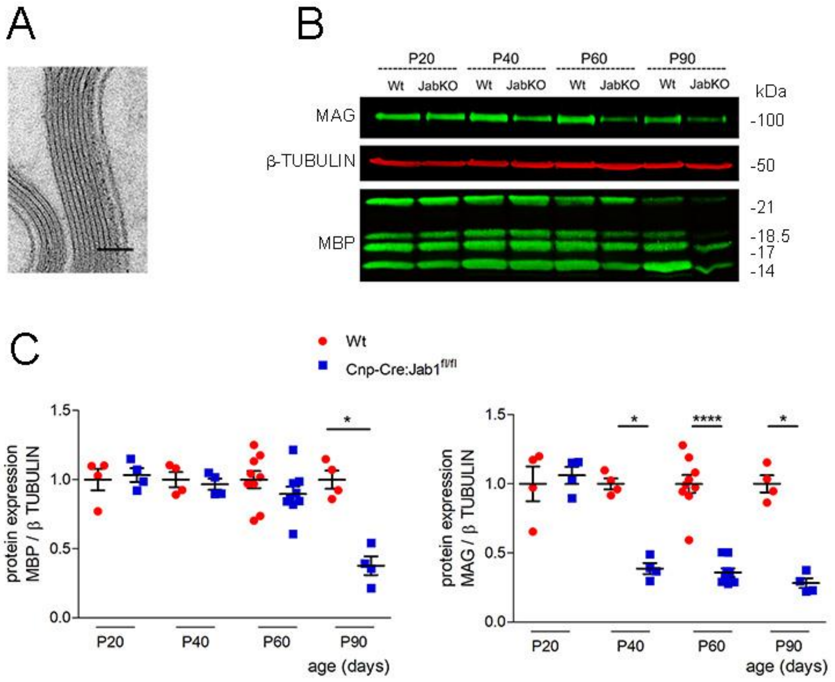

Supplemental Figure 2. Complementary signs of demyelination in mutant mice.
(**A**) Electron micrograph showing compact myelin in P20 *Cnp-Cre:Jab1$^{fl/fl}$* optic nerve. (**B**) Western blot analysis of the optic nerve homogenate from *Wt* and *Cnp-Cre:Jab1$^{fl/fl}$* mice at different ages and (**C**) quantification, showing progressive reduction of myelin proteins MBP (4 bands) and MAG (represented as a ratio of MBP/β-tubulin and MAG/β-tubulin; *p<0.05, ****p<0.0001; n=4 to 9; Two-tailed nonparametric Mann–Whitney U-test). Scale Bar, (**A**) 66 nm.

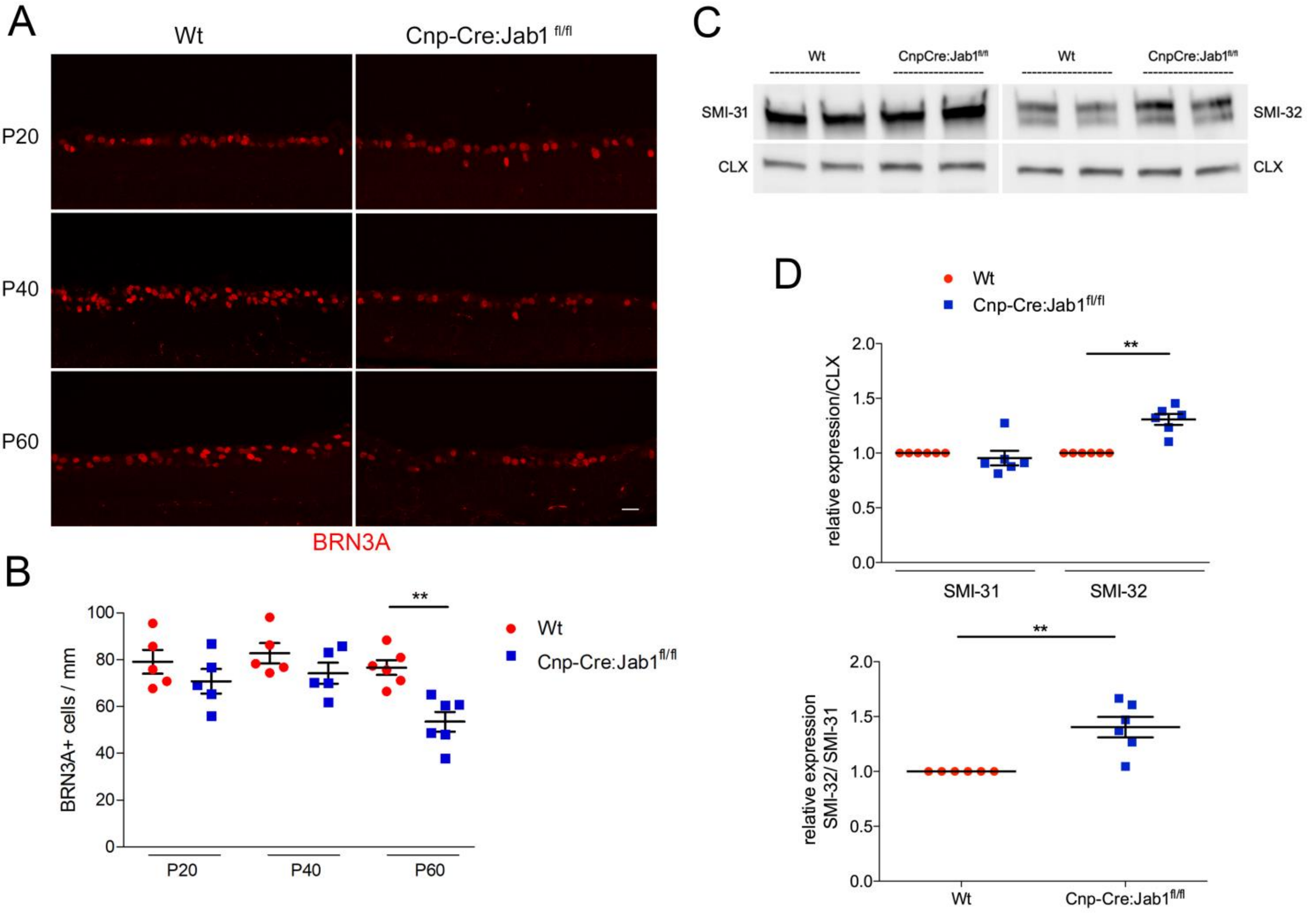

Supplemental Figure 3. Complementary signs of neurodegeneration in mutant mice.
(**A**) Confocal immunolabelling for BRN3A in the retina of Wt and *Cnp-Cre:Jab1*$^{fl/fl}$ mice at different ages. (**B**) Quantification of BNR3A-positive ganglion cells in the retina showing a significant reduction in mutant mice at P60 (**p<0,01; n=5 to 6; Two-tailed nonparametric Mann–Whitney U-test). (**C**) Western blot analysis for phosphorylated (SMI-31) and non-phosphorylated (SMI-32) neurofilaments of high molecular weight (NF-H) in the brain homogenate from P60 Wt and *Cnp-Cre:Jab1*$^{fl/fl}$ mice, and (**D**) quantification showing increased amount of SMI-32/SMI-31 ratio in mutant mice (**p<0.01; n=6; one sample two-tailed Student's t-test). Scale bar, (**A**) 50 µm

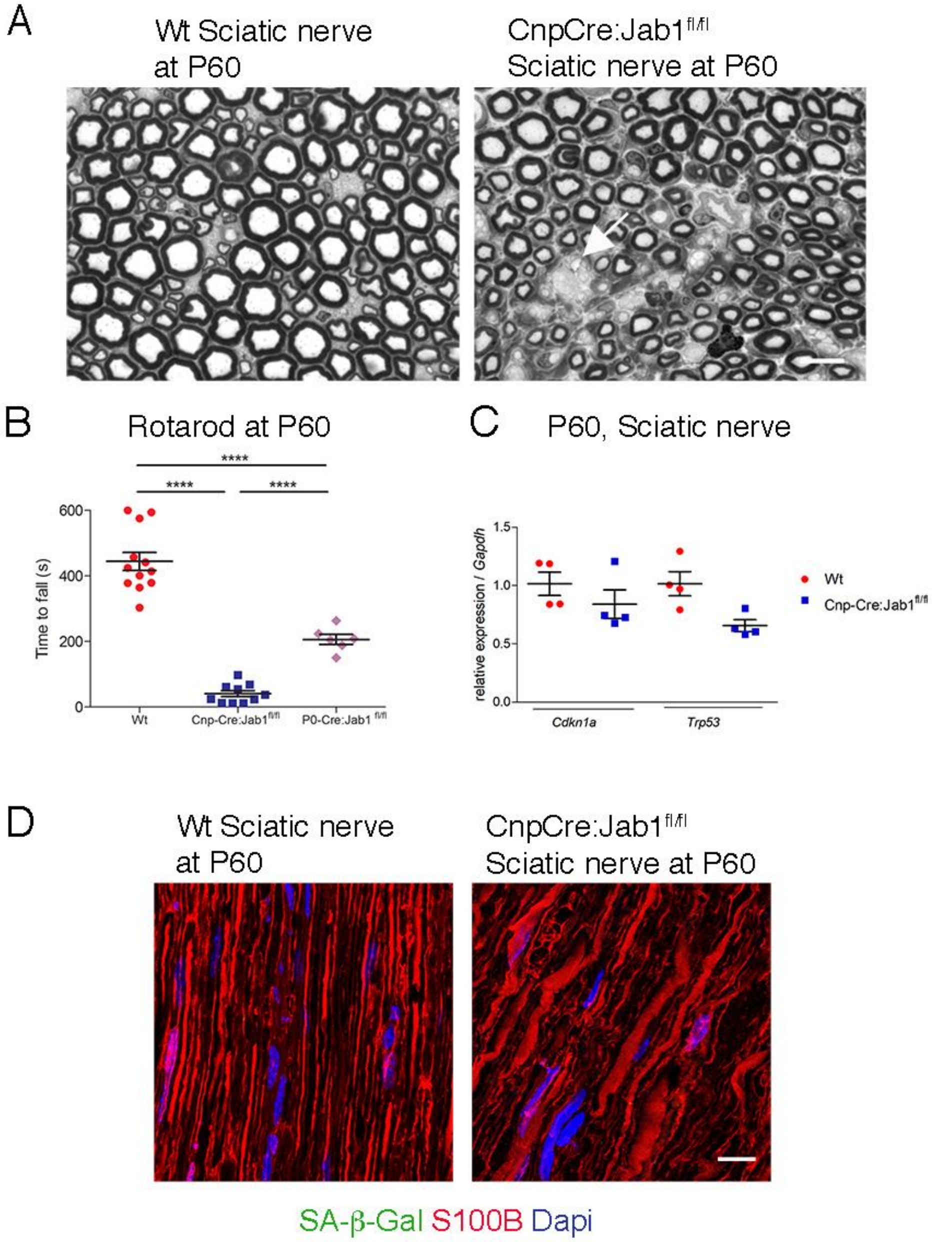

Supplemental Figure 4. Peripheral nerve involvement of mutant mice

(**A**) Light microscopy of sciatic nerve sections from *Wt* and *Cnp-Cre:Jab1*$^{fl/fl}$ mice at P60 showing hypomyelination and bundles of unsorted axons (arrow) in mutant sciatic nerves. (**B**) Rotarod analysis showing that motor deficits are significantly worsen in *Cnp-Cre* as respect to *P0-Cre* mutants (****$p<0.0001$; n= 6-11; One-way ANOVA with Bonferroni's multiple comparison test). (**C**) qPCR showing the expression of *Cdkn1a* (p21^{CIP1}) and *Trp53* (p53) in the Sciatic nerve of Wt and *Cnp-Cre:Jab1*$^{fl/fl}$ mice. (D) Confocal images showing no SA-β-Gal staining in mutant sciatic nerve. Scale bars, (**A**) and (**D**) 10 µm.

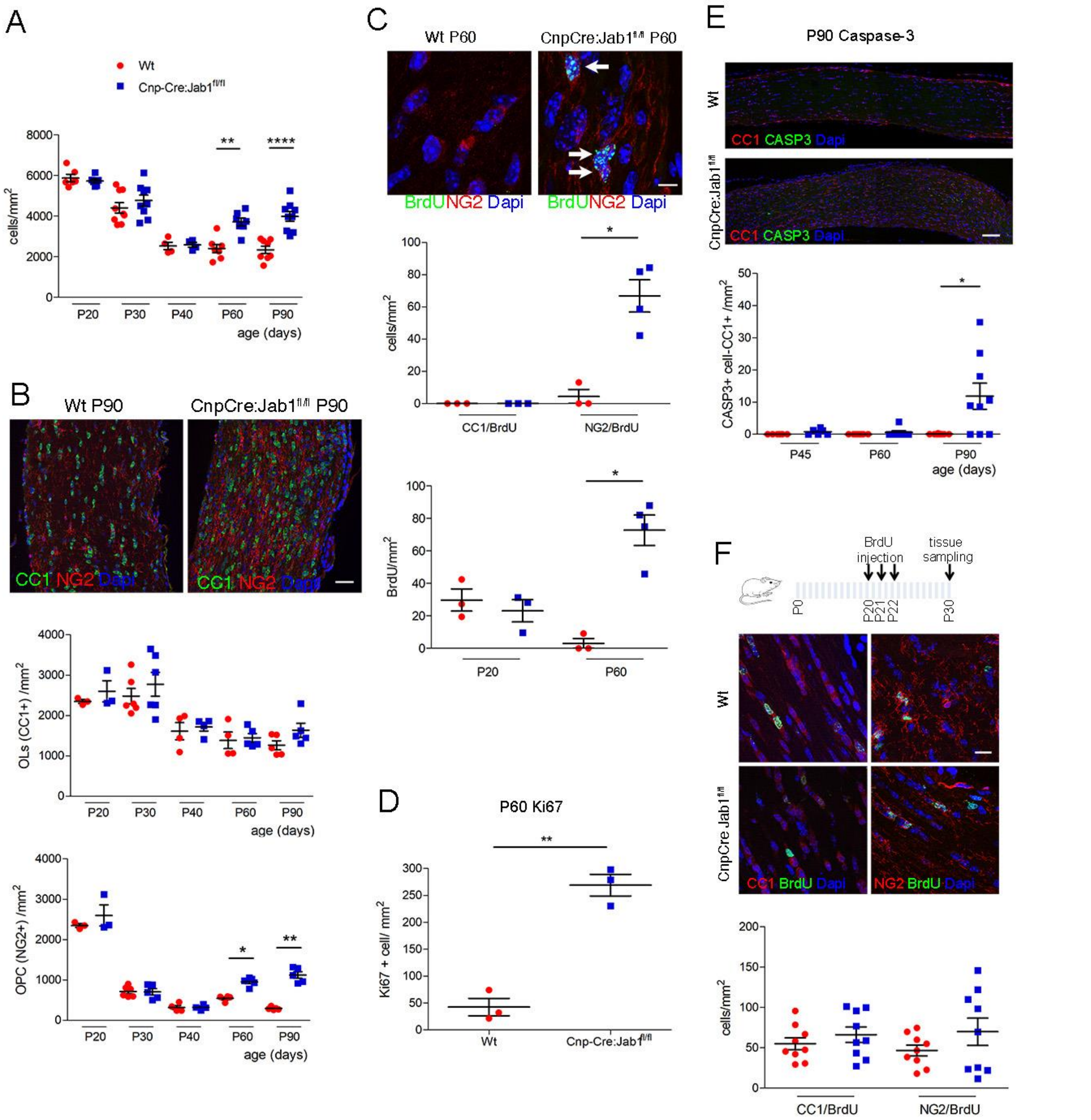

Supplemental Figure 5. Mutant oligodendrocyte proliferation, survival and differentiation
(**A**) Quantification of cell number (Dapi positive nuclei) in the optic nerve from *Wt* and *Cnp-Cre:Jab1*$^{fl/fl}$ mice at different ages (**p<0.01, ****p<0.0001; n=5 to 8; Two-tailed nonparametric Mann–Whitney U-test). (**B**) Confocal images of optic nerves from *Wt* and *Cnp-Cre:Jab1*$^{fl/fl}$ mice stained for CC1, NG2 and Dapi and relative quantification at different ages (*p<0.05, **p<0.01; n=3 to 5; Two-tailed nonparametric Mann–Whitney U-test). (**C**) Confocal images of optic nerves from *Wt* and *Cnp-Cre:Jab1*$^{fl/fl}$ mice stained for BrdU, NG2 and Dapi, 2 hours after BrdU injection; double positive OPCs are identified by arrows. Aside, quantification of BrdU$^+$ OPC (NG2$^+$), BrdU$^+$ mature oligodendrocytes (CC1$^+$) and total BrdU$^+$ cells at different ages (*p<0.05; n=3 to 4; Two-tailed nonparametric Mann–Whitney U-test). (**D**) Quantification of Ki67$^+$ cells in the optic nerve from *Wt* and *Cnp-Cre:Jab1*$^{fl/fl}$ mice (**p<0.01; n=3; Two-tailed nonparametric Mann–Whitney U-test). (**E**) Confocal images of optic nerves from *Wt* and *Cnp-Cre:Jab1*$^{fl/fl}$ mice stained for CASPASE-3 and oligodendrocyte marker (CC1) and relative quantification of CASPASE-3$^+$ oligodendrocytes at different ages (*p<0.05; n=7 to 9; Two-tailed nonparametric Mann–Whitney U-test). (**F**) Confocal immunolabelling of optic nerves from P30 *Wt* and *Jab1* mutant mice with anti-BrdU, CC1 or NG2, and quantification of CC1/BrdU or NG2/BrdU double positive cells showing similar numbers in *Wt* and *Jab1* mutant optic nerves (p=not significant; n=9; Two-tailed nonparametric Mann–Whitney U-test). Scale bars, (**B**) 40 µm, (**C**) 10 µm (**E**) 40 µm, (**F**) 10 µm.

P60 Optic nerve

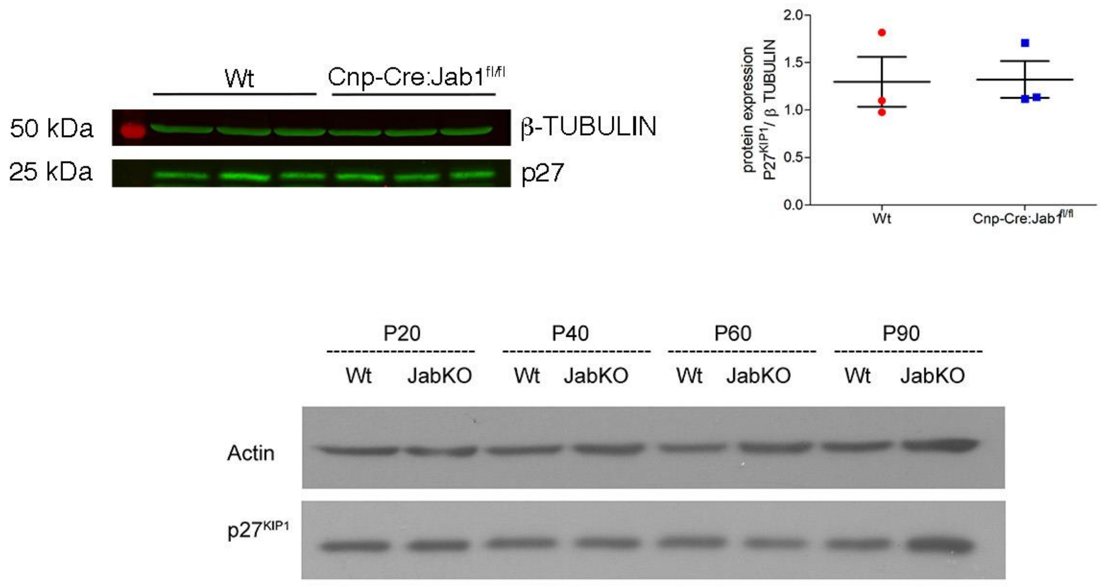

Supplemental Figure 6. Quantification of p27^{KIP1} in optic nerves

Western blot analysis (and quantification) for p27^{KIP1} in the optic nerve homogenate from *Wt* and *Cnp-Cre:Jab1$^{fl/fl}$* mice.

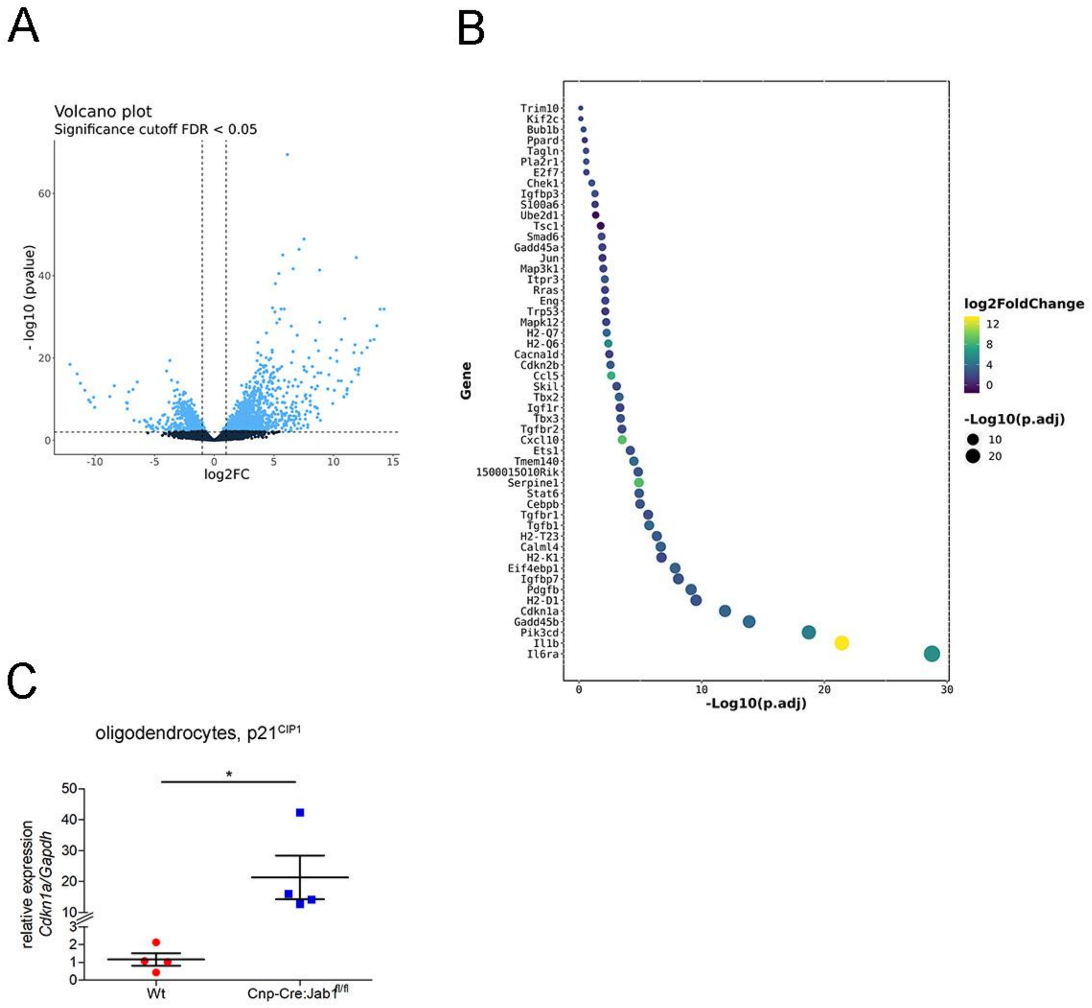

Supplemental Figure 7. FACS-sorted mutant oligodendrocytes express high levels of senescence and inflammatory genes

(**A**) Volcano plot representing significant down and up-regulated genes in mutant oligodendrocytes as compared to Wt as control. (**B**) Graphic representation of genes associated to senescence and inflammation significantly upregulated in mutant oligodendrocytes by RNA-seq analysis. (**C**) qPCR for *Cdkn1a* ($p21^{CIP1}$) in oligodendrocytes (*$p<0.05$; n=4; Two-tailed nonparametric Mann–Whitney U-test).

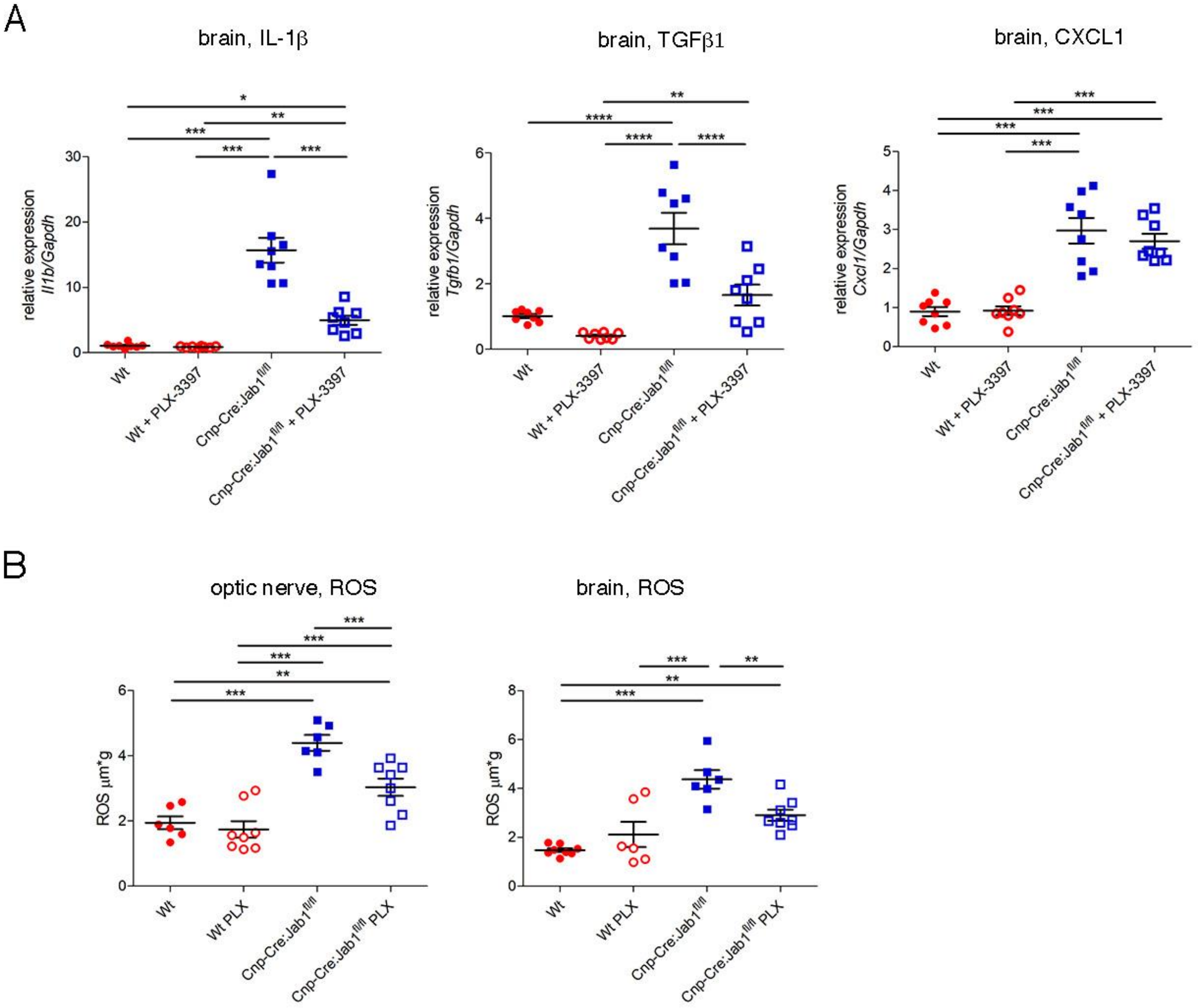

Supplemental Figure 8. Microglia depletion does not abrogate SASP and ROS elevation in the mutant mice

(**A**) Quantification of qPCR analysis for SASP in the brain homogenate of *Wt* and *Cnp-Cre:Jab1*$^{fl/fl}$ mice treated or not with PLX. (**B**) ROS quantification in corpus callosum and optic nerve from *Wt* and *Cnp-Cre:Jab1*$^{fl/fl}$ mice treated or not with PLX (*p<0.05, **p<0.01; ***p<0,001, ****p<0,0001; n= 6-8; One-way ANOVA with Bonferroni's multiple comparison test).

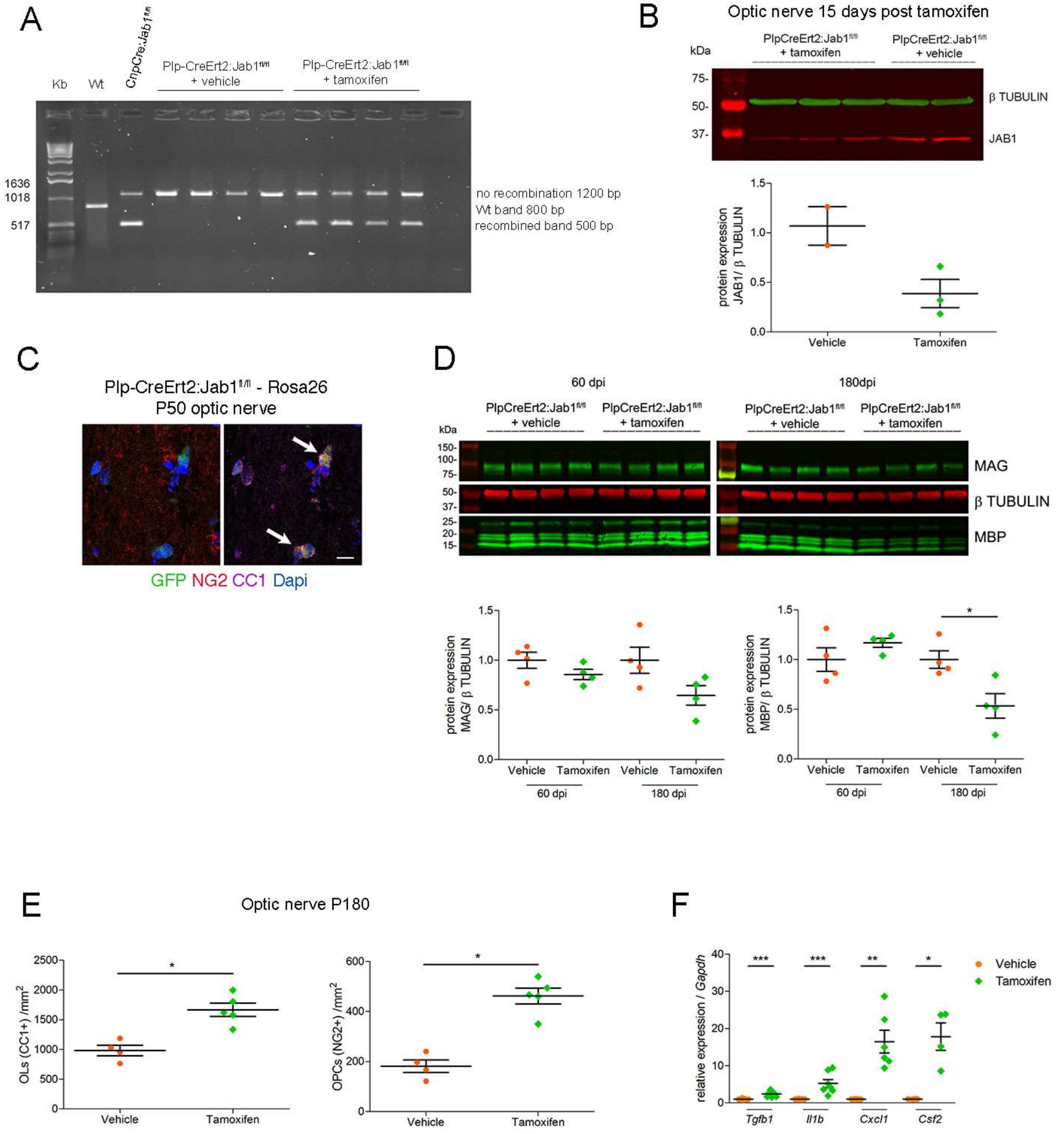

Supplemental Figure 9. Complementary characterization of Plp-CreERT2:Jab1$^{fl/fl}$ mice

(**A**) Genotyping showing the recombinant band in the optic nerve homogenate from tamoxifen-treated *Plp-CreERT2:Jab1$^{fl/fl}$* mice. (**B**) Western blot analysis of the optic nerve homogenate from *Plp-CreERT2:Jab1$^{fl/fl}$* mice treated with tamoxifen or vehicle and relative quantification showing reduced JAB1 protein in the recombined optic nerves (n=2 to 3; Two-tailed nonparametric Mann–Whitney U-test). (**C**) Confocal images of the optic nerve from P50 tamoxifen- *Plp-CreERT2:Jab1$^{fl/fl}$*:Rosa26 mice stained for GFP, CC1, NG2 and Dapi; recombined oligondendrocytes (CC1^{+}; GFP^{+}) are labelled by arrows. (**D**) Western blot analysis and quantification of the optic nerve homogenate from *Plp-CreERT2:Jab1$^{fl/fl}$* mice treated with tamoxifen or vehicle and stained for myelin proteins MAG and MBP at different ages; quantification is expressed as the ratio MAG/β-TUBULIN and MBP/β-TUBULIN (*p<0.05; n=4; Two-tailed nonparametric Mann–Whitney U-test). (**E**) Quantification of the number of oligodendrocytes (CC1+) and OPCs (NG2+) in the P180 optic nerve from *Plp-CreERT2:Jab1$^{fl/fl}$* mice treated with tamoxifen or vehicle (*p<0.05; n=4 Vehicle, n=5 Tamoxifen; Two-tailed nonparametric Mann–Whitney U-test). (**F**) Quantification of qPCR analysis for different SASP molecules in the P180 optic nerve homogenate from *Plp-CreERT2:Jab1$^{fl/fl}$* mice treated with tamoxifen or vehicle (*p<0.05, **p<0.01, ***p<0.001; n=4 to 7; Two-tailed nonparametric Mann–Whitney U-test). Scale bar, (**C**) 10 µm.

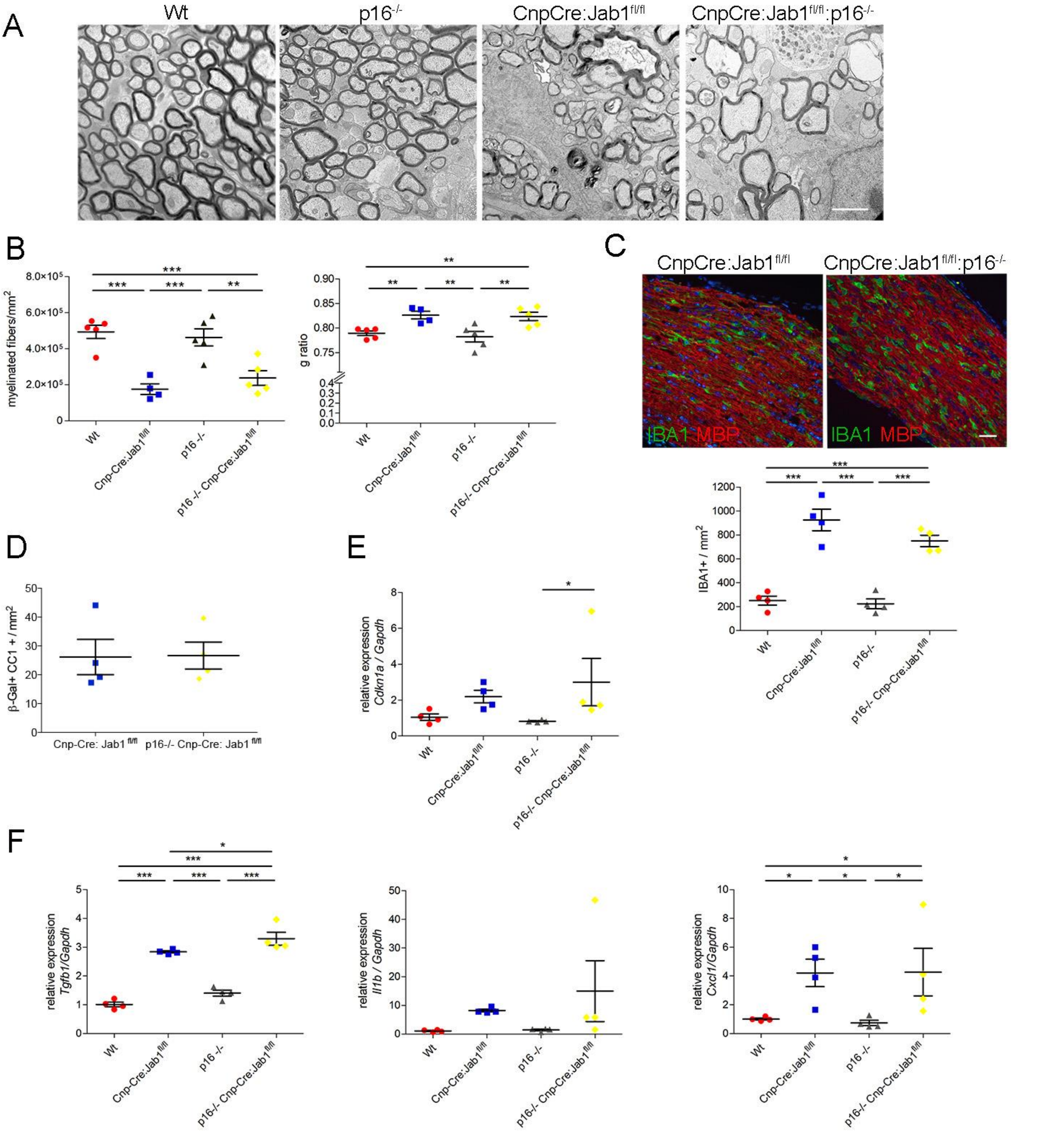

Supplemental Figure 10. Deletion of *p16^{INK4}* does not ameliorate the phenotype in Jab1 mutant mice.

(**A**) Electron micrographs of transverse sections of the optic nerve at P60 show similar demyelination and axonal loss in *Cnp-Cre:Jab1$^{fl/fl}$;p16$^{INK4-/-}$* as compared to *Cnp-Cre:Jab1$^{fl/fl}$*. (**B**) Quantification of the number of myelinated fibers and of g-ratio (**p<0.01, ***p<0,001; n=4; One-way ANOVA with Bonferroni's multiple comparison test). (**C**) Confocal images showing IBA1- inflammatory infiltrate in optic nerve and quantification (***p<0.001; n=4; One-way ANOVA with Bonferroni's multiple comparison test). (**D**) Quantification of SA-β-gal activity in CC1^{+} oligodendrocytes in the optic nerve of *Cnp-Cre:Jab1$^{fl/fl}$;p16$^{INK4-/-}$* as compared to *Cnp-Cre:Jab1$^{fl/fl}$* mice (p=not significant; n=4; Two-tailed nonparametric Mann–Whitney U-test). (**E**) qPCR analysis for *Cdnk1a* (p21^{CIP1}) in the optic nerve of four groups of mice (*p<0.05; n=4; One-way ANOVA with Bonferroni's multiple comparison test). (**F**) qPCR for SASP in the optic nerve of the four group of mice (*p<0.05, ***p<0,001; n=4; One-way ANOVA with Bonferroni's multiple comparison test). Scale bars, (**A**) 2 µm, (**C**) 40 µm.

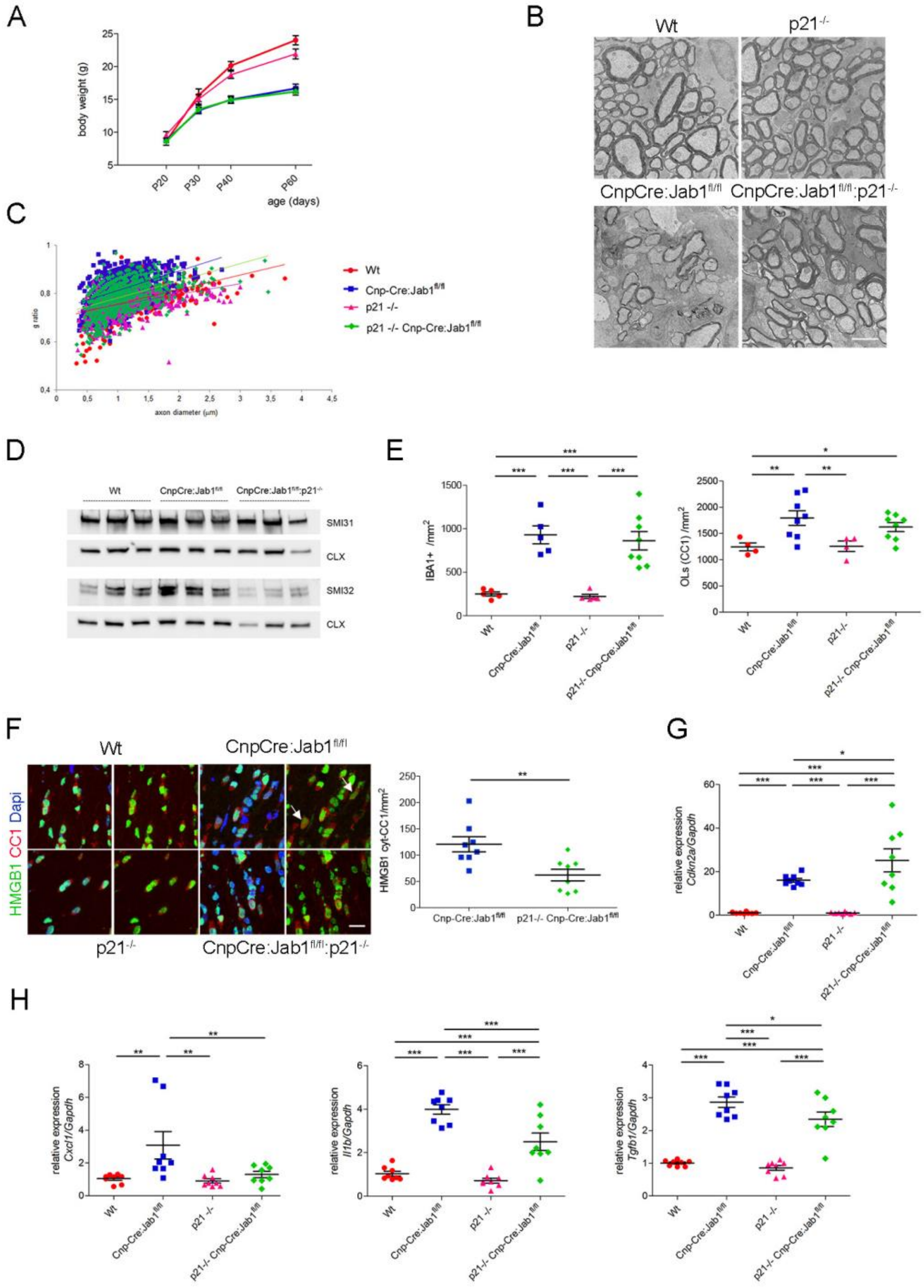

Supplemental Figure 11. Complementary findings in tissues of *Cnp-Cre:Jab1$^{fl/fl}$;p21$^{CIP1-/-}$* mice.
(**A**) Mouse body weight at different ages. (**B**) Electron micrographs of transverse sections of the optic nerve at P60 showing amelioration of demyelination and axonal loss in *Cnp-Cre:Jab1$^{fl/fl}$;p21$^{CIP1-/-}$* as compared to *Cnp-Cre:Jab1$^{fl/fl}$* mice. (**C**) Representative g-ratio versus axon diameter scatter plot (and linear regression lines) of the myelinated fibers in the optic nerve of the 4 groups of mice showing values for *Cnp-Cre:Jab1$^{fl/fl}$;p21$^{CIP1-/-}$* mice in between Wt and *Cnp-Cre:Jab1$^{fl/fl}$* mice (5 mice per group; at least 2000 fibers per mouse). (**D**) Western blot analysis for phosphorylated (SMI-31) and non-phosphorylated (SMI-32) neurofilaments of high molecular weight (NF-H) in the brain homogenate from P60 Wt and *Cnp-Cre:Jab1$^{fl/fl}$* and *Cnp-Cre:Jab1$^{fl/fl}$;p21$^{CIP1-/-}$* mice (quantification in Figure 8E). (**E**) Quantification of IBA1^{+} cell and CC1^{+} oligodendrocytes in the optic nerve of the 4 groups of mice (*$p<0.05$, **$p<0.01$, ***$p<0{,}001$; n=4; One-way ANOVA with Bonferroni's multiple comparison test). (**F**) Confocal images for HMGB1 in P60 optic nerves of the 4 groups of mice showing nuclear localization in all the oligodendrocytes in Wt and p21$^{-/-}$ mice; in *Cnp-Cre:Jab1$^{fl/fl}$* mice, many oligodendrocytes showed HMGB1 cytoplasm localization (arrows) while this was significantly reduced in *Cnp-Cre:Jab1$^{fl/fl}$;p21$^{CIP1-/-}$* mice (aside quantification; **$p<0.01$, n=8; Two-tailed nonparametric Mann–Whitney U-test). (**G**) qPCR for *Cdkn2a* (p16^{INK4}) in P60 optic nerve homogenate showing similar levels in *Cnp-Cre:Jab1$^{fl/fl}$* and *Cnp-Cre:Jab1$^{fl/fl}$;p21$^{CIP1-/-}$* mice (*$p<0.05$, ***$p<0.001$; n=8; One-way ANOVA with Bonferroni's multiple comparison test). (**H**) qPCR for SASP proinflammatory factors *Cxcl1*, *Il1b* and *Tgfb1* in P60 optic nerve homogenate, showing significant reduction in *Cnp-Cre:Jab1$^{fl/fl}$;p21$^{CIP1-/-}$* as compared to *Cnp-Cre:Jab1$^{fl/fl}$* mice (*$p<0.05$, **$p<0.01$, ***$p<0.001$; n=8; One-way ANOVA with Bonferroni's multiple comparison test). Scale bar, (**B**) 2 µm, (**F**) 20 µm

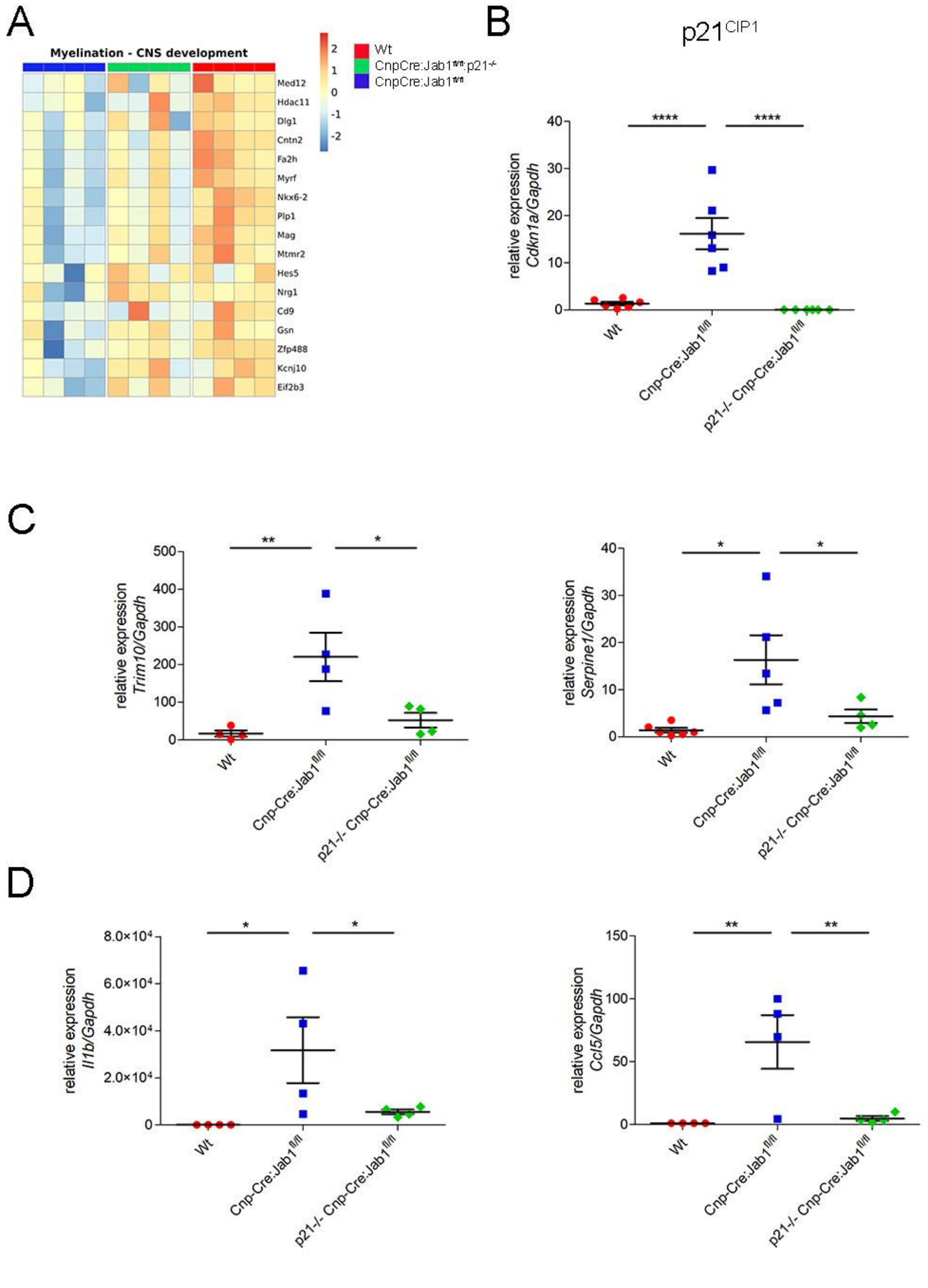

Supplemental Figure 12. Complementary finding in FACS-sorted oligodendrocytes of *Cnp-Cre:Jab1$^{fl/fl}$;p21$^{CIP1-/-}$* mice

(**A**) Heat map representing the expression values of key genes for myelination differentially expressed in Wt, *Cnp-Cre:Jab1$^{fl/fl}$* and *Cnp-Cre:Jab1$^{fl/fl}$;p21$^{CIP1-/-}$* FACS-sorted O1$^+$ oligodendrocytes. (**B**) qPCR for *Cdkn1a* in FACS-sorted O1$^+$ oligodendrocytes showing the absence of p21^{CIP1} in *Cnp-Cre:Jab1$^{fl/fl}$;p21$^{CIP1-/-}$* mice. Confirmatory qPCR for senescence (**C**) and SASP (**D**) genes differentially expressed in FACS-sorted O1$^+$ oligodendrocytes (*p<0.05, **p<0.01,; n=4; One-way ANOVA with Bonferroni’s multiple comparison test).

Supplemental Table 1. List of the differentially expressed genes in O1+ sorted oligodendrocytes with FDR p-value≤0.05 between Cnp-Cre:Jab1$^{fl/fl}$ (KO) and Control (Wt)
See excel file Table 3-DGE analysis KO_vs_Ctrl

Supplemental Table 2. List of the differentially expressed genes in O1+ sorted oligodendrocytes with FDR p-value ≤0.05 between Cnp-Cre:Jab1$^{fl/fl}$ p21^{CIP1}-/- (dKO) and Control (Wt)
See excel file Table 4-DGE analysis dKO_vs_Ctrl

Supplemental Table 3. List of the differentially expressed genes in O1+ sorted oligodendrocytes with FDR p-value ≤0.05 between Cnp-Cre:Jab1$^{fl/fl}$ p21^{CIP1}-/- (dKO) and Cnp-Cre:Jab1$^{fl/fl}$ (KO)
See excel file Table 5-DGE analysis dKO_vs_KO

Supplemental Table 4, Animal Models

Animals	Specie	Strain	Provider	Backcrosses	Sex	Age	References
COPS5/Jab1$^{fl/fl}$	Ms	C57Bl/6J	Dr. R. Pardi (San Raffaele Scietific Institute, Milan, Italy)	>F10	M/F	P20 P30 P40 P50 P60 P90	Panattoni et al, 2008
Rosa26-EYFP	Ms	129X1/SvJ	Jackson Laboratory (stock No. 006148)	>F10	M/F	P30	Srinivas et al, 2001
Cnp1-Cre	Ms	C57Bl/6J	Dr. K.A. Nave (Max Planck Institute of Experimental Medicine, Gottingen, Germany)	>F10	M/F	P20 P30 P40 P50 P60 P90	Lappe-Siefke et al, 2003
PLP1- creERT2	Ms	B6.D2 F1/J	Dr. U. Suter (Institute of Cell Biology, Zurich, Switzerland)	>F10	M/F	P120 P180	Leone et al, 2003
B6.129S4-Ccr2^{tm1Ifc}/J	Ms	129S4/SvJae	Jackson Laboratory (stock No. 004999)	>F10	M/F	P50	Boring et al, 1997
B6.129S6(Cg)-Cdkn1a^{tm1Led}/J	Ms	129S6/SvEvTac	Jackson Laboratory (stock No. 016565)	>F10	M/F	P40 P60	Deng et al, 1995
B6.Cg-Tg(S100b-verbB)4496Waw Cdkn2a^{tm1Rdp}/Nci	Ms	C57Bl/6J	NCI-Frederick MMHCC Repository, MD 21701, USA (Code 01XD3)	>F10	M/F	P60	Sharpless et al, 2001

Ms=Mouse; M= Male; F= Female; P=Post natal day

Supplemental Table 5, *PCR primer sequences*

Gene	Primer	Label	Primer sequence 5’ → 3’
Floxed Jab1 allele	Jab1 d1	InII1 F	GGT CAG AAA GCT AGG CCT AAG AAG G
	Jab1 d2	ExII1 R	GGC ATG CAT CAC CAT TTT CAG TAG
Deletad Jab1 allele	Jab1 d1	InII1 F	GGT CAG AAA GCT AGG CCT AAG AAG G
	Jab1 int	InII R	GGG CTT AGG AAT GCC AAG C
Cnp1	Cnp E3 sense	Ex3 F	GCC TTC AAA CTG TCC ATC TC
	Cnp E3 R	R	CCC AGC CCT TTT ATT ACC AC
	Cnp 3 puro	PGKfor	CAT AGC CTG AAG AAC GAG A
Plp1	Plp1	Int1 F	TGG ACA GCT GGG ACA AAG TAA GC
	Plp2	Cre-R	CGT TGC ATC GAC CGG TAA TGC AGG C
EYFP-Rosa26	Rosa26 892	Mut R	AAG ACC GCG AAG AGT TTG TC
	Rosa26 545	F	AAA GTC GCT CTG AGT TGT TAT
	Rosa26 546	R	GGA GCG GGA GAA ATG GAT ATG
Ccr2	Ccr2 A	Mut F	CTC GTG CTT TAC GGT ATC GC
	Ccr2 B	R	ATG GCG CAA GGC TAT TTG
	Ccr2 C	WT F	GCC CAC AAA ACC AAA GAT GA
Cdkn2a	I001	F	GTG ATC CCT CTA CTT TTT CTT CTG ACT T
	I002	WT R	CGG AAC GCA AATATC GCA C
	I003	Mut R	GAG ACT AGT GAG ACG TGC TAC TTC CA
Cdkn1a	12427	F	GTT GTC CTC GCC CTC ATC TA
	12428	WT R	GCC TAT GTT GGG AAA CCA GA
	12429	Mut R	CTG TCC ATC TGC ACG AGA CTA

F= Forward, R=Reverse, WT= wild type, Mut= Mutant, In= intron, Ex= Exon

Supplemental Table 6, Primary Antibody

ANTIBODY	CLONE	SPECIE	CATALOG #	COMPANY	DILUITION
ACTIN		pRb	A2066	Sigma-Aldrich	1:2000 (WB)
APC (Ab-7)	CC-1	mMS	OP80	Calbiochem	1:20 (IF)
BrdU	BMC 9318	mMs	11 170 376 001	Roche	1:20 (IF)
BRN3A	14°6	mMS	sc-8429	Santa Cruz Biotechnology	1:100 (IF)
Calnexin		pRb	C4731	Sigma-Aldrich	1:5000 (WB)
CD11b-PE-Cy7	M1/70	mRt	552850	BD Pharmigen™	1:300 (FACS)
CD11c-PE	N418	mHm	117307	BioLegend	1:300 (FACS)
CD45R-Pacific Blue	RA3-6B2	mRt	558108	BD Pharmigen™	1:300 (FACS)
Cleaved Caspase-3	Asp175	pRb	9661	Cell Signaling	1:100 (IF)
DNA-PKcs (pS2056)		pRb	ab18192	abcam	1:200 (IF)
GFAP	G2.2B10	mRt	13-0300	Invitrogen™	1:100 (IF)
GFP		pCh	GFP-1020	Aves Labs	1:500 (IF)
Phospho-H2A.X (Ser139)		pRb	2577	Cell Signaling	1:50 (IF)
HMGB1		pRb	ab18256	abcam	1:200 (IF)
HMGB1	EPR3507	mRb	Ab79823	abcam	1:10000 (WB)
IBA1		pRb	019-19741	Wako	1:200 (IF)
JAB1		pRb	J3020	Sigma-Aldrich	1:1000 (WB); 1:500 (IF)
Ki67		pRb	NCL-Ki67p	Novocastra™	1:100 (IHC)
Ly6C-FITC	AL-21	mRt	553104	BD Pharmigen™	1:200 (FACS)
Ly6G-PerPc™-Cy5.5	1A8	mRt	560602	BD Pharmigen™	1:200 (FACS)
MAG		pRb		Gift of James L. Salzer, NYU School of Medicine, New York, New York 10016, USA.	1:1000 (WB)
MBP	a.a.82-87	mRt	MAB386	Merck	1:1000 (WB); 1:50 (IF)
Neurofilaments H, Non-Phosphorylated	SMI32	mMS	SMI32-R	Covance	1:2000 (WB)
Neurofilaments, Phosphorylated	SMI31	mMS	SMI31-P	Covance	1:5000 (WB)
NG2		pRb		Gift of Bill Stallcup, Sandorf Burnham Medical Research, La Jolla, California 92037, USA	1:200 (IF)
O1-eFluor660	O1	mMs	50-6506-82	eBioscience™	1:100 (FACS)
OLIG2		pRb	ab136253	abcam	1:500 (IF)
OLIG2	3C9	mMs	SAB1404798	Sigma-Aldrich	1:50 (IF)
p27$^{(Kip1)}$	57/Kip1/p27	mMs	610241	BD Transduction Laboratories™	1:1000 (WB)
b-TUBULIN	TUB2.1	mMs	T4026	Sigma-Aldrich	1:2000 (WB)

m=monoclonal, p=polyclonal, Ch= Chicken, Gt=Goat, Hm=Hamster, Ms=Mouse, Rb=Rabbit, Rt=Rat

Supplemental Table 7, Secondary Antibody for IHC and WB

ANTIBODY	CATALOG #	COMPANY	DILUITION
FITC Goat anti-Mouse	1034-02	SouthernBiotech	1:100
TRITC Goat anti Mouse IgG_{2b}	1090-03	SouthernBiotech	1:100
TRITC Donkey anti-Mouse	715-025-151	Jackson InnunoResearch Labs	1:100
AF 488 Goat anti-Mouse IgG1	A21121	Thermo Fisher Scientific	1:1000
AF 488 Goat anti-Mouse IgG	A11001	Thermo Fisher Scientific	1:1000
FITC Donkey ant-Rat	712-095-150	Jackson InnunoResearch Labs	1:100
TRITC Donkey ant-Rat	712-025-153	Jackson InnunoResearch Labs	1:100
FITC Goat anti-Rabbit	4030-02	SouthernBiotech	1:100
TRITC Goat anti-Rabbit	4030-03	SouthernBiotech	1:100
AF 594 Goat anti-Rabbit IgG	A11012	Thermo Fisher Scientific	1:1000
Anti-Rabbit IgG - peroxidase	A0545	Sigma-Aldrich	1:10000
Anti-Mouse TrueBlot Ig HRP	18-8817-33	Rockland antibody & assay	1:10000
IRDye 800CW goat anti Mouse	926-32210	Li-Cor Biosciences	1:10000
IRDye 800CW goat anti Rabbit	926-32211	Li-Cor Biosciences	1:10000
IRDye 800CW goat anti Rat	926-32219	Li-Cor Biosciences	1:10000
IRDye 680LT goat anti Mouse	926-68020	Li-Cor Biosciences	1:10000
IRDye 680 goat anti Rabbit	926-32221	Li-Cor Biosciences	1:10000
IRDye 680RW goat anti Rat	926-68076	Li-Cor Biosciences	1:10000
